# Region-specific drivers of CSF mobility measured with MRI in humans

**DOI:** 10.1038/s41593-025-02073-3

**Published:** 2025-10-14

**Authors:** Lydiane Hirschler, Bobby A. Runderkamp, Andreas Decker, Thijs W. van Harten, Paul Scheyhing, Philipp Ehses, Léonie Petitclerc, Julia Layer, Eberhard Pracht, Bram F. Coolen, Wietske van der Zwaag, Tony Stöcker, Philipp Vollmuth, Daniel Paech, Alexander Effland, Marianne A. A. van Walderveen, Alexander Radbruch, Mark A. van Buchem, Gabor C. Petzold, Susanne J. van Veluw, Matthan W. A. Caan, Katerina Deike, Matthias J. P. van Osch

**Affiliations:** 1 C.J. Gorter MRI Center, Department of Radiology, Leiden University Medical Center, Leiden, the Netherlands; 2 Department of Radiology and Nuclear Medicine, Amsterdam University Medical Center, University of Amsterdam, Amsterdam, the Netherlands; 3 German Center for Neurodegenerative Diseases (DZNE), Bonn, Germany; 4 Department of Neurology, Massachusetts General Hospital, Harvard Medical School, Boston, MA USA; 5 Department of Neuroradiology, University Hospital Bonn, Bonn, Germany; 6 Department of Vascular Neurology, University Hospital Bonn, Bonn, Germany; 7 Department of Biomedical Engineering and Physics, Amsterdam University Medical Center, University of Amsterdam, Amsterdam, the Netherlands; 8 Spinoza Centre for Neuroimaging, Royal Netherlands Academy of Arts and Sciences, Amsterdam, the Netherlands; 9 Computational Cognitive Neuroscience and Neuroimaging, Netherlands Institute for Neuroscience, KNAW, Amsterdam, the Netherlands; 10 Department of Physics and Astronomy, University of Bonn, Bonn, Germany; 11 Division for Computational Radiology & Clinical AI (CCIBonn.ai), Clinic for Neuroradiology, University Hospital Bonn, Bonn, Germany; 12 Division for Medical Image Computing, German Cancer Research Center, Heidelberg, Germany; 13 Department of Radiology, Brigham and Women’s Hospital, Harvard Medical School, Boston, MA USA; 14 Institute of Applied Mathematics, University Bonn, Bonn, Germany; 15 Athinoula A. Martinos Center for Biomedical Imaging, Massachusetts General Hospital, Charlestown, MA USA

**Keywords:** Magnetic resonance imaging, Neuro-vascular interactions, Neurodegeneration

## Abstract

Many neurological diseases are characterized by the accumulation of toxic proteins in the brain. This accumulation has been associated with improper clearance from the parenchyma. Recent discoveries highlighted perivascular spaces, which are cerebrospinal fluid (CSF)-filled spaces, as the channels of brain clearance. The forces driving CSF mobility within perivascular spaces are still debated. Here we present a noninvasive, CSF-specific magnetic resonance imaging technique (CSF-Selective T_2_-prepared REadout with Acceleration and Mobility-encoding) that enables detailed in vivo measurement of CSF mobility in humans, down to the level of perivascular spaces located around penetrating vessels, which is close to protein production sites. We find region-specific drivers of CSF mobility and demonstrate that CSF mobility can be increased by entraining vasomotion. Furthermore, we find region-specific CSF mobility alterations in patients with cerebral amyloid angiopathy, a brain disorder associated with clearance impairment. The availability of this technique opens up avenues to investigate the impact of CSF-mediated clearance in neurodegeneration and sleep.

## Abstract (web summary)

Brain clearance mechanisms are challenging to visualize in humans. Using magnetic resonance imaging, the authors noninvasively mapped cerebrospinal fluid motion across the brain, showing region-specific drivers in healthy participants and altered dynamics in cerebral amyloid angiopathy.

## Main

Because of its high metabolic rate, the brain produces large quantities of proteins, whose abnormal accumulation is involved in a number of pathologies and neurodegenerative disorders. However, unlike other organs in the body, the brain tissue lacks a classic lymphatic system to transport excess soluble proteins it produces (‘waste’) out of the brain (for example, to lymph nodes or dural lymphatic vessels). The true nature of brain clearance mechanisms has eluded characterization for centuries, until the recent uptick in interest in the topic^1,2^. The use of microscopy in rodents^3–5^ and invasive intrathecal injections in humans^6,7^ has allowed researchers to unravel and describe many aspects of new brain clearance pathways and more subtle physiological processes; their results have opened up new questions and debate. While we know that brain clearance processes must involve cerebrospinal fluid (CSF) as the main carrier of ‘waste’ products, probably along pathways surrounding small blood vessels called perivascular spaces (PVS)^8^, the exact anatomical pathway(s), driving force(s) and physiological processes involved in CSF-mediated brain clearance remain unresolved^1,9,10^. Some studies suggested the presence of an active mechanism driving CSF flow along PVS (including the glymphatic^3^ and intramural periarterial drainage^11^ theories), whereas others propose that perivascular clearance mainly occurs via more passive mixing mechanisms^9,12^. In all proposed mechanisms, CSF mobility in PVS would be facilitated by physiological motion processes, such as cardiac pulsations^4^, respiration^13^ or vasomotion^5^. As such, it has been suggested that these drivers of motion can propel soluble ‘waste’ products from the PVS up toward the pial surface, where bulk flow assures further egress.

Studying CSF-mediated brain clearance and its driving forces is of particular importance because clearance failure has been implicated in the accumulation of toxic proteins in the brain, such as amyloid-β and tau. A plethora of neurological diseases like Alzheimer’s disease^14^, cerebral amyloid angiopathy (CAA)^15^, traumatic brain injury^16^ and ischemic stroke^17^ are associated with brain clearance deficiencies. CAA, especially, is a common small-vessel disease and leading cause of hemorrhagic stroke and dementia in older individuals, which is characterized by the accumulation of amyloid-β in the vessel wall, possibly because of impaired CSF-mediated amyloid-β clearance^15,18^. CAA frequently co-occurs with Alzheimer’s pathology and is associated with increased risk of developing amyloid-related imaging abnormalities in the context of anti-amyloid immunotherapy^15^. Notably, in a rat model of CAA, the mobility of the CSF in the subarachnoid space (SAS) surrounding large arteries was recently found to be increased^19^. This was accompanied by a reduction of volume of tracer transport to the brain tissue, altogether suggesting that CSF would bypass the brain tissue because of amyloid-β deposits. However, it is currently unknown whether these recent findings in rodents also translate to humans with CAA. Therefore, a better understanding of CSF-mediated brain clearance and its driving forces is urgent as it would provide crucial new avenues not only to elucidate the pathophysiology of these neurological diseases, but also toward new targets for efficient therapeutic strategies to slow or stop disease progression.

Unfortunately, current knowledge of CSF-mediated brain clearance mechanisms is mostly derived from experimental studies performed in rodents, which are associated with substantial limitations. First, these studies tend to use techniques that introduce perturbations to the very system they aim to characterize, such as euthanasia before measurement, which may result in the collapse of essential structures for brain clearance^4^, anesthesia, which interferes with hemodynamics and also probably with brain clearance, or invasive imaging techniques^3–5,20^ like cranial windows and injection of fluorescent dyes, which may induce local pressure changes and thereby affect physiological CSF motion. Second, it is unclear how experimental findings in rodents translate to humans given the inherent differences in brain size and physiological parameters between species^2^. For example, cardiac frequency is about 7–12 times slower in humans compared to mice^21^, whereas vasomotion frequency is comparable, centered around 0.1 Hz. This could influence the relative contributions of the suggested driving forces of CSF flow, namely the cardiac cycle, respiration and vasomotion. Moreover, to the best of our knowledge, current in vivo studies only focus on motion of the CSF in the SAS and in the ventricular system^22–26^, lacking the spatial resolution to investigate CSF mobility in PVS around penetrating vessels, which are believed to be channels along which soluble ‘waste’ products clear out of the brain.

A noninvasive method that measures CSF mobility at a high spatial resolution is needed to further understand perivascular brain clearance mechanisms in humans, allowing the assessment of CSF mobility directly where clearance is thought to occur. This would pave the way for studying CSF-mediated brain clearance in larger sample sizes, patient cohorts and in longitudinal follow-up studies. Magnetic resonance imaging (MRI) at a high magnetic field strength (7 Tesla) is an excellent modality for such a noninvasive imaging strategy because it provides the necessary resolution to image the PVS and has the added benefit of easily differentiating the CSF from other brain tissues or fluids by exploiting its specific magnetic properties.

In this study, we present a noninvasive, high-resolution and CSF-specific MRI technique that allows the characterization of CSF mobility, even in PVS around penetrating vessels. We also investigate and compare the influence of the cardiac cycle, respiratory cycle and vasomotion as driving forces for CSF mobility. Lastly, we apply this imaging technique in patients with CAA in a pilot study to explore its potential to improve our understanding of neurodegenerative diseases.

## Results

### High-resolution imaging of CSF mobility is achieved using CSF-STREAM

**Fig. 1:**
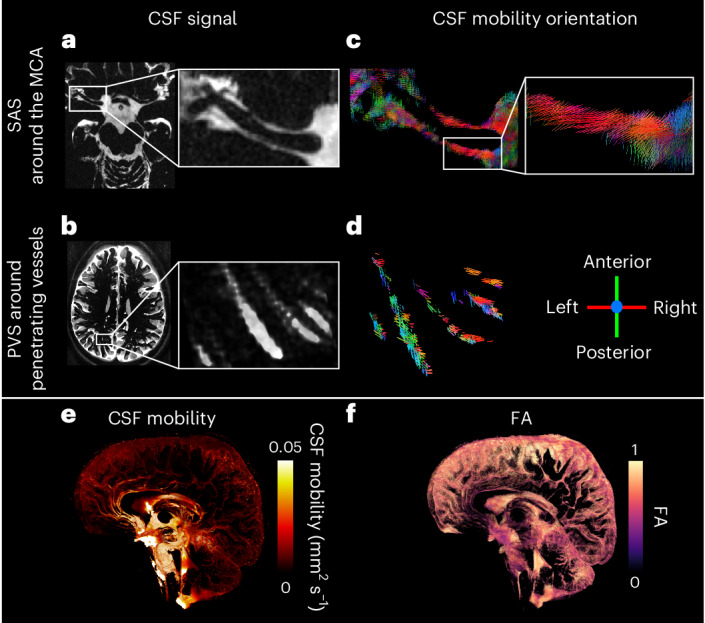
CSF signal and CSF mobility characteristics using CSF-STREAM. **a**,**b**, CSF signal, measured using the non-motion-sensitized reference scan, in the SAS around the MCA (**a**) and in PVS around penetrating vessels in one representative individual (**b**). **c**,**d**, Principal orientation of CSF mobility in the SAS around the MCA (**c**), including a zoomed area on one branch, and in PVS of penetrating vessels (**d**); **c**,**d** are from the same ROIs as **a** and **b**. The line colors reflect the orientation of CSF mobility: red indicates a left-to-right orientation, green an anterior-posterior orientation and blue a head-to-feet orientation. **e**,**f**, Volume rendering of a CSF mobility map (in mm^2^ s^−1^) (**e**) and an FA map in one representative individual (**f**).

**Fig. 2:**
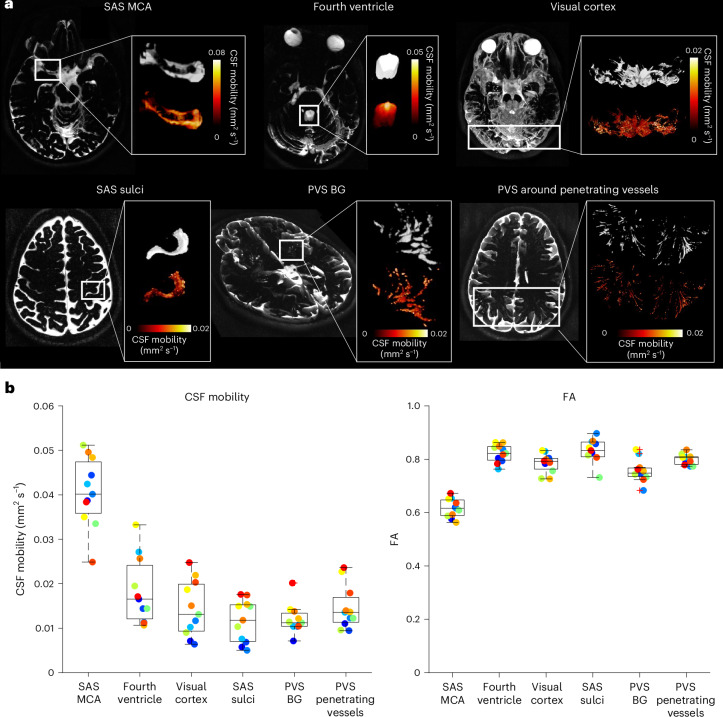
Regional CSF mobility and FA using CSF-STREAM. **a**, Example of the location of the ROIs in one representative individual: SAS around the MCA, fourth ventricle, CSF around the visual cortex, SAS of the motor cortex sulci, PVS in the BG and PVS surrounding the penetrating vessels. In each insert, the extracted volume rendering of the ROI is shown (top) next to the CSF mobility (in mm^2^ s^−1^) volume rendering within the ROI (bottom). **b**, Average CSF mobility (in mm^2^ s^−1^) (left) and FA (right) in the different ROIs in 11 individuals. Each point represents the average value over the voxels in each ROI per individual (one color per individual). In each box plot, the central line indicates the median, and the bottom and top edges of the box indicate the 25th and 75th percentiles, respectively. The whiskers extend to the most extreme data points not considered outliers; outliers are plotted individually using the ‘+’ marker symbol. Source data

**Extended Data Fig. 1:**
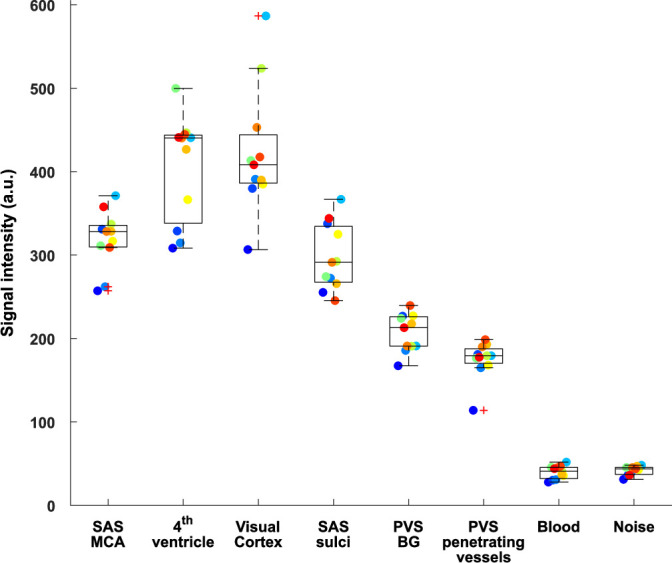
Mean signal intensity in different regions of interest. Average signal intensity (in a.u.) measured on the non-motion-sensitized reference scan in eight regions of interest of eleven individuals. Each point represents the average value over voxels in each ROI per individual (one color per individual). On each boxplot, the central line indicates the median, and the bottom and top edges of the box indicate the 25^th^ and 75^th^ percentiles, respectively. The whiskers extend to the most extreme data points not considered outliers, and the outliers are plotted individually using the ‘+‘ marker symbol. Source data

**Extended Data Fig. 2:**
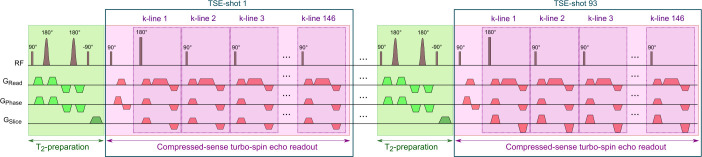
Sequence diagram of one CSF-STREAM sub-scan. Example of the first and last turbo-spin-echo (TSE)-shots. The whole subscan consists of 93 TSE-shots containing each 146 k-lines. Each TSE-shot is preceded by a T_2_-preparation in which motion-sensitizing gradients can be added to encode CSF mobility. In total, a CSF-STREAM scan consists of seven subscans: one without motion-sensitizing gradients and six with gradients applied in different, orthogonal directions. In this figure, motion-sensitizing gradients are applied in the read and phase directions.

The whole-brain CSF signal was visualized in 20 healthy, younger individuals (aged 33 ± 12 years, 16 females, four males) at rest using ultra-high-field (7 Tesla) MRI with a T_2_-prepared high-resolution (0.45-mm isotropic voxel size) accelerated readout with a long echo time (TE) (Figs. 1a,b and 2a, Extended Data Fig. 1 and Supplementary Video 1). Importantly, the signal originating from the blood and brain tissue was suppressed (that is, not significantly different from the noise level), such that the CSF signal was successfully isolated (Extended Data Fig. 1). The introduction of motion-sensitizing gradients of 3.5 mm s^−1^ in the T_2_ preparation module, which during repeated measurements encode mobility in six orthogonal directions, allowed calculation of a tensor from which the CSF mobility, fractional anisotropy (FA) and principal CSF mobility orientation were computed (Fig. 1). CSF mobility is measured and calculated in a similar way as an apparent diffusion coefficient acquired at a very low *b*-value, making the MRI sequence more sensitive to flow than diffusion^27^. The term ‘mobility’ is used as opposed to ‘flow’ or ‘diffusion’ to accentuate that the dephasing underlying the signal attenuation is caused by either slow flow, laminar flow or by back-and-forth motion of the CSF, or a combination of all processes^22,27^. Altogether, the proposed CSF-Selective T_2_-prepared REadout with Acceleration and Mobility-encoding (CSF-STREAM) provides a fully noninvasive technique to quantitatively measure the mobility of the CSF at an unprecedented high resolution: from the ventricles to the SAS around large vessels, down to small PVS in the basal ganglia (BG) and around penetrating vessels (Fig. 2 and Extended Data Fig. 2).

In the SAS around the middle cerebral artery (MCA) and in PVS, the CSF mainly moves along the vessel as represented by principal vector orientation (Fig. 1c,d). The FA in PVS (Fig. 2b) was high, further substantiating that the CSF preferentially moves along one orientation. CSF mobility was higher at the base of the brain than at the brain surface (Figs. 1e and 2b). From the selected regions of interest (ROIs) (Fig. 2b), CSF mobility was highest in the SAS around the MCA (0.041 ± 0.008 mm^2^ s^−1^) and three times lower in the PVS of the BG and of penetrating vessels (0.012 ± 0.003 mm^2^ s^−1^ and 0.015 ± 0.005 mm^2^ s^−1^, respectively).

### Region-specific effects of cardiac and respiratory cycles on CSF mobility

**Fig. 3:**
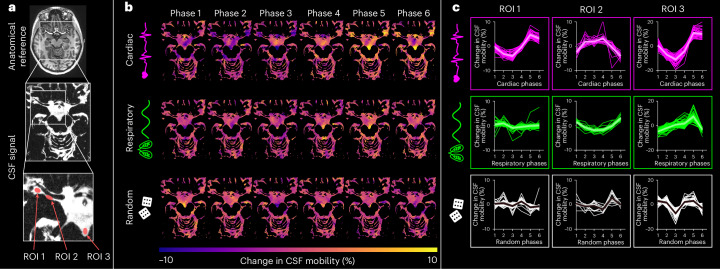
Change in CSF mobility across driving forces in large CSF spaces around the circle of Willis. **a**, Anatomical location and inserts of the CSF signals corresponding to **b** and **c**. **b**, Maps of change in CSF mobility from the mean value over phases (%) across the cardiac (top), respiratory (middle) and random (bottom) cycles in one representative individual. **c**, Voxel-wise CSF mobility changes in three ROIs shown in the bottom insert in **a**. Each colored line represents the signal of an individual voxel within the ROI; the thicker line represents the mean value over the voxels in that ROI and the shaded area the confidence interval (CI) over the voxels. Source data

**Fig. 4:**
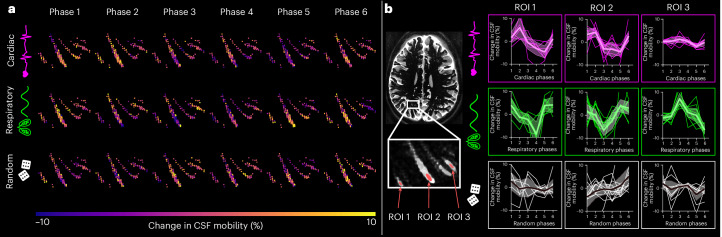
Change in CSF mobility across driving forces in PVS around penetrating vessels. **a**, Maps of change in CSF mobility from the mean value over phases (%) across the cardiac (top), respiratory (middle) and random (bottom) cycles in one representative individual. **b**, Voxel-wise changes in CSF mobility in three ROIs shown in the insert. Each colored line represents the signal of an individual voxel within the ROI; the thick line represents the mean value over the voxels in that ROI and the shaded area the CI over the voxels. Source data

**Fig. 5:**
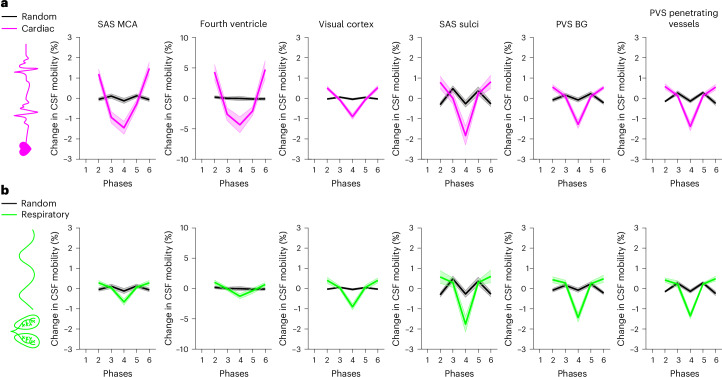
Regional comparison of change in CSF mobility across driving forces. **a**,**b**, Change in CSF mobility from the mean value over phases (%) across the cardiac cycle (pink) (**a**), respiratory cycle (green) (**b**) and random cycle (gray) (**a**,**b**) in six ROIs in 11 individuals. Note that the *y* axis range is different for the ROI of the fourth ventricle. Each line represents the mean over individuals; the shaded error areas represent the CIs of s.d. × 1.96 (√*n*)^−1^ (*n* = 11). Source data

**Fig. 6:**
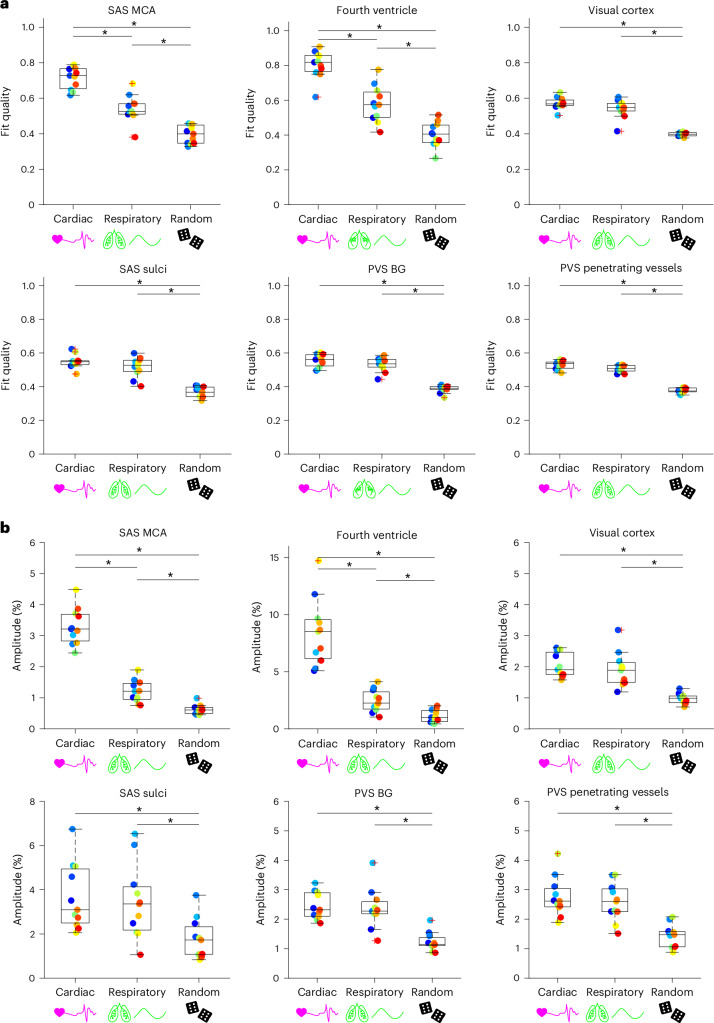
Regional comparison of CSF mobility voxel-wise fit quality and amplitude change across driving forces. **a**,**b**, Comparison between the effect of the cardiac, respiratory and random binning on the fit quality (**a**) and amplitude (**b**) of the change in CSF mobility (%) in 11 individuals. Note that the *y* axis range is different for the fourth ventricle and SAS sulci ROIs in **b**. Each data point represents the value per individual in an ROI. The single asterisks indicate significant differences with *P* < 0.01 using a two-sided Wilcoxon signed-rank test applied when a Friedman test was significant. In each box plot, the central line indicates the median and the bottom and top edges of the box indicate the 25th and 75th percentiles, respectively. The whiskers extend to the most extreme data points not considered outliers; the outliers are plotted individually using the ‘+’ marker symbol. Source data

**Extended Data Fig. 3:**
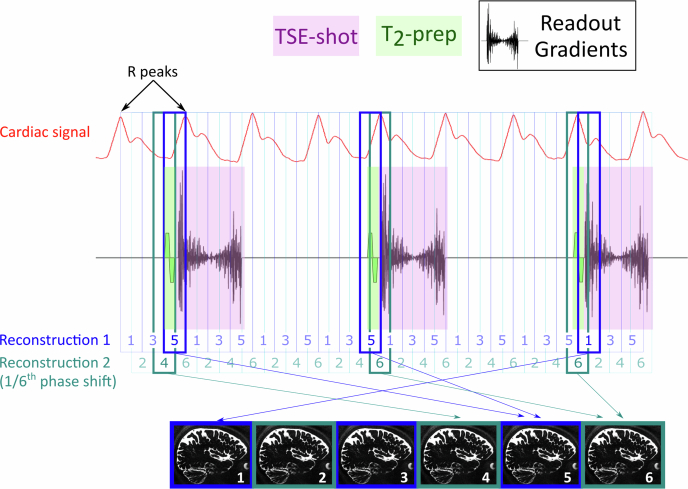
Retrospective binning. Schematic representation of the binning in cardiac phases with reference to the T_2_-preparation preceding each turbo-spin-echo (TSE)-shot. This scheme only represents 3 out of the 93 shots. The retrospective binning in 6 phases is performed in two steps: k-space is binned twice in 3 phases, with a 1/6^th^ phase shift between the two steps. The T_2_-preparation preceding each TSE-shot is taken as reference and the signal in the subsequent TSE-shot is considered to be dependent on this specific cardiac phase.

**Extended Data Fig. 4:**
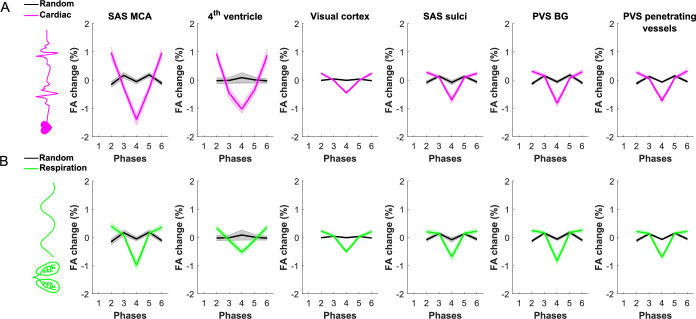
Regional comparison of fractional anisotropy change across driving forces. FA change from the mean value over phases (in %) across (**A**) the cardiac cycle (pink), (**B**) respiratory cycle (green) and (**A**&**B**) random phases (grey) in six regions of interest in eleven individuals. Each line represents the mean over individuals and the shaded error areas represent confidence intervals of SD×1.96/√n (n = 11). Source data

Next, the influence of the driving forces of CSF mobility was assessed in 11 individuals by studying the effect of the cardiac and respiratory cycles on CSF mobility, as these are proposed to be driving forces of CSF mobility^2^. After retrospective binning of the *k*-space to the recordings of external physiological sensors (Extended Data Fig. 3), we observed that CSF mobility and FA were indeed fluctuating across the cardiac and respiratory cycles in an oscillatory manner (Figs. 3–5 and Extended Data Fig. 4). We found that in selected large CSF spaces located at the base of the brain, namely the SAS around the MCA (SAS-MCA) and the fourth ventricle, the cardiac cycle was associated with significantly larger CSF mobility oscillations than respiration. This is shown by the CSF mobility changes across phases averaged over individuals in Fig. 5 and the significantly better fit quality to a sinusoid (SAS-MCA: 0.7 ± 0.06 for cardiac versus 0.5 ± 0.08 for respiration, *P* = 0.002; fourth ventricle: 0.8 ± 0.08 for cardiac versus 0.6 ± 0.1 for respiration, *P* = 0.002; two-sided Wilcoxon signed-rank test) and change in amplitude of CSF mobility (SAS-MCA: 3.2 ± 0.6% for cardiac versus 1.2 ± 0.4% for respiration, *P* < 0.001; fourth ventricle: 8.4 ± 2.9% for cardiac versus 2.5 ± 1.0% for respiration, *P* < 0.001; two-sided Wilcoxon signed-rank tests), as shown in Fig. 6. In contrast, in the smaller PVS in the BG and around penetrating vessels, CSF mobility was driven by similar contributions of the cardiac and respiratory cycles (Figs. 5 and 6): the cardiac cycle induced a change in amplitude of CSF mobility of 2.5 ± 0.5% in BG PVS and 2.8 ± 0.7% in PVS around penetrating vessels, whereas respiration led to 2.4 ± 0.7% (BG PVS) and 2.6 ± 0.6% (penetrating PVS) change in amplitude of CSF mobility.

**Extended Data Fig. 5:**
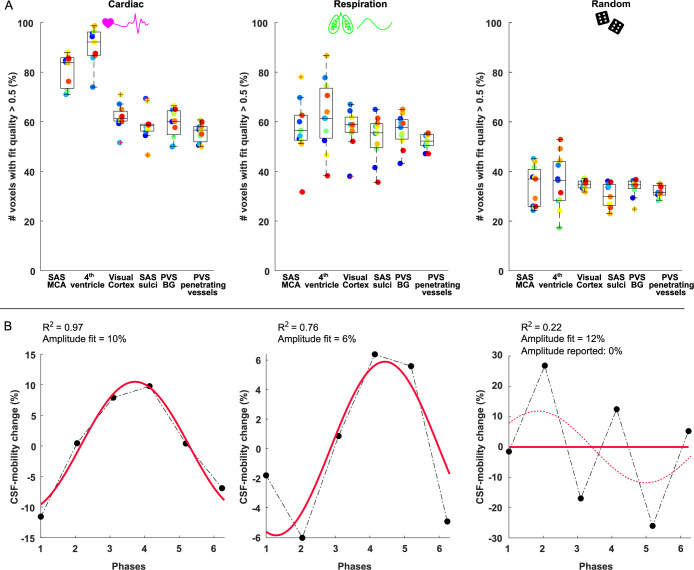
Voxel-wise fit. (**A**) Percentage of voxels showing coherent CSF mobility changes across phases (that is R^2^ > 0.5 when fitting a sinusoidal model) relative to the total amount of voxels in the ROI, in eleven individuals. Values are shown for cardiac (left), respiratory (middle) and random binning (right) in six ROIs. Each datapoint represents the value per individual in a region of interest. On each boxplot, the central line indicates the median, and the bottom and top edges of the box indicate the 25^th^ and 75^th^ percentiles, respectively. The whiskers extend to the most extreme data points not considered outliers, and the outliers are plotted individually using the ‘+‘ marker symbol. (**B**) Example of the voxel-wise sinusoidal fit (full line, in red) applied to the signal across phases (black dashed line with dots) in three different voxels. The associated fit quality and amplitude are shown above each graph. In the most right panel, the reported amplitude would have be 12%, even though the fit quality to the sinusoid is very low (R^2^ = 0.22, the corresponding fit is the red dashed line) and does not represent well the CSF mobility change over phases. In that case, an amplitude of 0 is reported instead, as shown by the flat red line to better represent the absence of coupling. Source data

Our method of retrospective binning did not artificially generate the observed changes in CSF mobility because random binning did not result in a clear oscillatory pattern across phases (Fig. 5): CSF mobility varies across random phases in a zig-zag-like fashion with, for example, several dips and peaks within one cycle, indicating a noisier pattern than that observed across cardiac and respiratory phases. This can also be concluded quantitatively from the fit quality, fit amplitude and the amount of voxels with coherent changes across phases (that is, voxels with *R*^2^ > 0.5), which were all significantly lower for random ordering compared to binning based on cardiac or respiratory traces (Fig. 6 and Extended Data Fig. 5). Moreover, spatially coherent patterns (for example, a right-to-left symmetry) in CSF mobility fluctuations can be observed for cardiac and respiratory binning but not for random ordering (Fig. 3 and Supplementary Video 2), providing further confidence that these oscillations are indeed originating from physiological pulsations and are not an artificial result of the reconstruction step.

### Entraining vasomotion drives CSF mobility

**Fig. 7:**
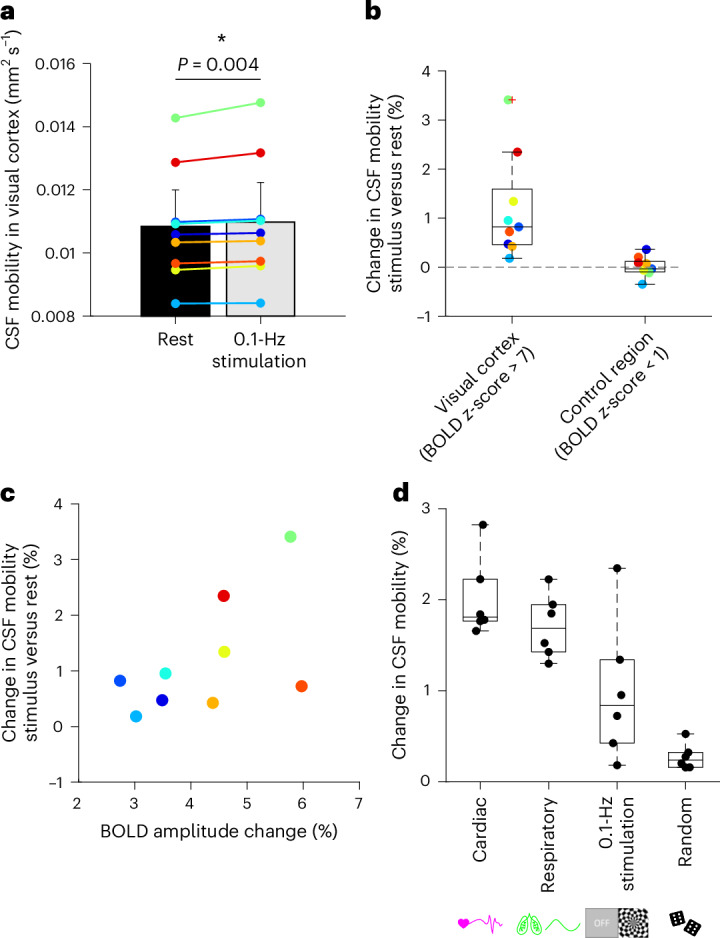
Effects of entrained vasomotion on CSF mobility and comparison to other driving forces. **a**, Average ± s.d. × 1.96 (√*n*)^−1^ CSF mobility (in mm^2^ s^−1^) at rest (black) and in the presence (gray) of a 0.1-Hz visual stimulation to entrain vasomotion in nine individuals. Each color represents the value in one individual. The asterisk indicates significant (*P* = 0.004) changes between the two conditions using a two-sided Wilcoxon signed-rank test. **b**, Change in CSF mobility (%) induced by the 0.1-Hz visual stimulation compared to rest in the visual cortex (defined as the region where the BOLD *z*-score was > 7) and in a control region (where the BOLD *z*-score was < 1) in nine individuals. **c**, Change in CSF mobility (%) in the visual cortex versus the BOLD amplitude change (%) in nine individuals. **d**, Change in CSF mobility (%) induced by different driving forces (cardiac, respiratory, visual stimulation and random) in six individuals who participated in both studies. In each box plot, the central line indicates the median and the bottom and top edges of the box indicate the 25th and 75th percentiles, respectively. The whiskers extend to the most extreme data points not considered outliers; the outliers are plotted individually using the ‘+’ marker symbol. Source data

Along with the cardiac and respiratory cycles, vasomotion has been proposed as one of the driving forces for the CSF. Unfortunately, there is no external physiological sensor to detect vasomotion that could be used for retrospective binning. A previous study in rodents showed that a 0.1-Hz visual stimulation could entrain vasomotion and lead to faster clearance of fluorescent tracers in the mouse brain^5^. Moreover, a recent study showed that visual stimulation can enhance CSF flow in the fourth ventricle^25^. To investigate whether visual stimulation also drives local CSF mobility in the SAS and PVS of the visual area in humans, a flashing checkerboard stimulus was shown to nine individuals while measuring CSF mobility. Entraining vasomotion by means of 0.1-Hz visual stimulation significantly increased CSF mobility in the visual cortex by 1.2% on average (range = 0.2–3.4%, two-sided Wilcoxon signed-rank test *P* = 0.004; Fig. 7a,b), compared to a control region outside the stimulated area (average difference in control region = 0.01%, range = −0.35 to 0.36%, *n* = 9, two-sided Wilcoxon signed-rank test *P* = 0.82). Note that the ‘visual cortex’ region consists of the CSF within the activated area in response to the visual stimulation (Methods). To investigate whether the spread in increase in CSF mobility across individuals originated from different responses to visual stimulation, we calculated the effect of visual stimulation on evoked vascular reactivity by quantifying the blood oxygenation level-dependent (BOLD) change in signal amplitude. A Bayesian correlation analysis gave anecdotal evidence for a positive correlation between BOLD amplitude and change in CSF mobility (BF_0+_ = 2.3; Fig. 7c), suggesting that the vessel wall displacement induced by visual stimulation potentially leads to the increase in CSF mobility^28^. The CSF mobility increase induced by entraining vasomotion approaches the amplitude change driven by the cardiac and respiratory cycles (Fig. 7d).

### Regional alterations of CSF mobility and FA in patients with CAA

**Fig. 8:**
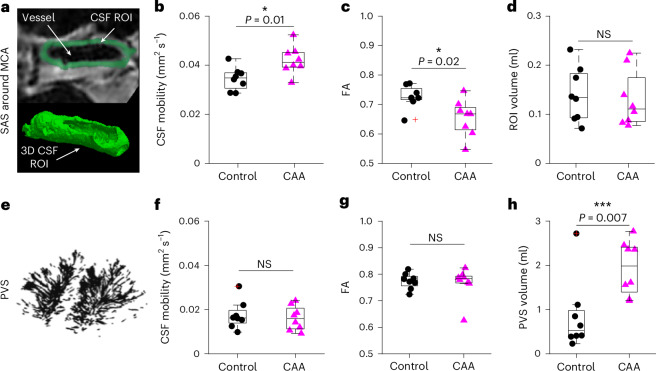
CSF-STREAM in patients with CAA versus healthy controls. **a**, Example of a 1-mm CSF rim in the SAS around the MCA. **b**, CSF mobility was significantly increased (*P* = 0.01, two-sided Mann–Whitney *U*-test). **c**, FA was significantly decreased (*P* = 0.02, two-sided Mann–Whitney *U*-test) in the 1-mm-thick SAS around the MCA of patients with CAA (pink) versus healthy controls (black). **d**, ROI volume around the MCA in controls and patients with CAA (*P* = 0.72, two-sided Mann–Whitney *U*-test). **e**, Example of PVS segmentation around penetrating vessels in the CSO. **f**,**g**, No significant change in CSF mobility (*P* = 0.88, two-sided Mann–Whitney *U*-test) (**f**) nor FA (*P* = 0.80, two-sided Mann–Whitney *U*-test) (**g**) was found in PVS. **h**, The PVS volume was significantly increased (*P* = 0.007, two-sided Mann–Whitney *U*-test) in patients with CAA. Each data point represents the value per individual (*n* = 8 controls and *n* = 8 patients with CAA) in an ROI. In each box plot, the central line indicates the median and the bottom and top edges of the box indicate the 25th and 75th percentiles, respectively. The whiskers extend to the most extreme data points not considered outliers; the outliers are plotted individually using the ‘+’ marker symbol. NS, not significant. Source data

**Extended Data Fig. 6:**
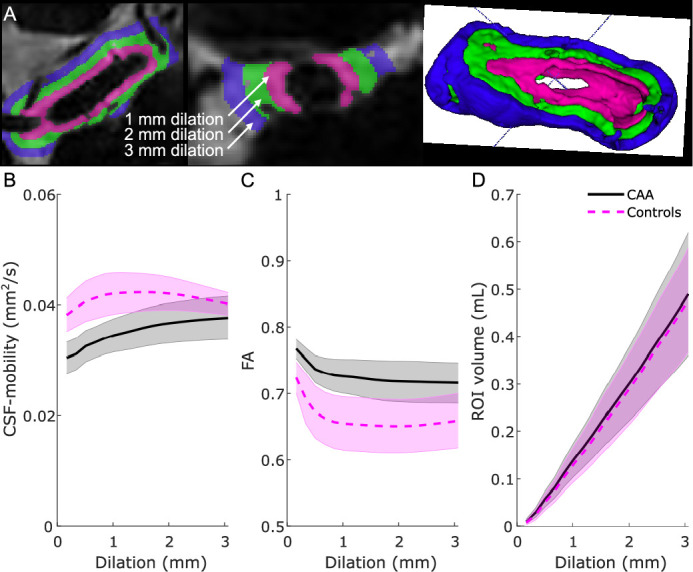
CSF mobility, FA and ROI volume as a function of the distance to the middle cerebral artery (MCA). (**A**) Example of dilations around the middle cerebral artery of 1 mm (magenta), 2 mm (green) and 3 mm (blue) containing CSF. (**B**) CSF mobility, (**C**) FA and (**D**) ROI volume across dilation width in CAA patients (pink, dashed line) and healthy controls (black, full line). Each line represents the mean over individuals and the shaded error areas represent confidence intervals of SD×1.96/√n (n = 8 per group). Source data

Lastly, to show the potential of CSF-STREAM to improve our understanding of neurodegenerative diseases, we applied the MRI sequence in eight patients with a clinical diagnosis of sporadic CAA, and in eight age-matched and sex-matched healthy controls (Fig. 8). We found a 20% increase in CSF mobility in the SAS closely surrounding the MCA of patients with CAA compared to healthy controls (CSF mobility_CAA_ = 0.042 ± 0.006 mm^2^ s^−1^, CSF mobility_Control_ = 0.035 ± 0.005 mm^2^ s^−1^, *P* = 0.01, two-sided Mann–Whitney *U*-test; Fig. 8b). This increase in CSF mobility was accompanied by a 10% decrease in FA (FA_CAA_ = 0.65 ± 0.06, FA_Control_ = 0.73 ± 0.04, *P* = 0.02, two-sided Mann–Whitney *U*-test; Fig. 8c). Of note, this significant group difference was detectable in the SAS around the MCA up to a radius of approximately 1.7 mm around the vessel; beyond this distance, the difference diminished (Extended Data Fig. 6).

In PVS around penetrating vessels in the centrum semiovale (CSO), CSF mobility and FA were not significantly different between groups (CSF mobility_CAA_ = 0.016 ± 0.006 mm^2^ s^−1^, CSF mobility_Control_ = 0.017 ± 0.006 mm^2^ s^−1^, *P* ≥ 0.05, two-sided Mann–Whitney *U*-test, Fig. 8f,g), whereas a significantly increased PVS volume was found in patients with CAA compared to controls, as expected^29^ (Fig. 8h; *P* = 0.007, two-sided Mann–Whitney *U*-test). Age, sex, lifestyle factors and cognitive and physical health scores did not differ between both groups (Extended Data Table 1).

## Discussion

In this study, we measured and directly compared the effects induced by several driving forces not only in large CSF spaces such as the fourth ventricle or in the SAS at the brain surface, as shown by previous studies^4,24–26,30^, but also in smaller PVS around penetrating vessels, which are closer to waste production sites. Moreover, we provide in vivo evidence of altered CSF mobility in patients with CAA, a disease associated with brain clearance impairment.

A strength of our results in the younger, healthy cohort is that a direct comparison can be made between the cardiac, respiratory and random cycles because all three originate from the same dataset, which was reconstructed in different ways, thus capturing the individual in the same physiological state. Our study shows that driving forces for CSF mobility are region-specific. In large CSF spaces located at the base of the brain (SAS around the MCA and fourth ventricle), the cardiac cycle is a larger contributing driving force to CSF mobility compared to respiration. Although posture influences CSF flow^31^ and is different in humans (supine) than in rodents (prone), our findings are in line with previous findings in mice^4^ and humans^23,30^ and can be explained by the fact that during systole the brain expands because of increased filling of its entire arterial system, which leads to CSF flowing out of the skull to compensate for the increased brain volume. This flow reverses during diastole. Conversely, in PVS located around penetrating vessels more distally in the vascular tree where cardiac pulsations are probably dampened down, our results point to similar influence on CSF mobility fluctuations by cardiac and respiratory cycles. This finding in PVS suggests that both cardiac and respiratory rhythms are equally important driving forces for perivascular CSF mobility. This supports the notion of multiple sources promoting mixing within PVS^12^, rather than a single wave caused by a single physiological phenomenon (for example, only arterial pulsations) pushing CSF through PVS^4,11^. However, in our study, the six cardiac and respiratory phases originated from data collected over 40 min. Therefore, possible effects from different types of respiration (for example, single events of deep breaths) would be averaged out. It can be hypothesized that forced breathing or deep inspirations could further enhance CSF mobility fluctuations, as shown previously^24,30^.

Low-frequency (~0.1 Hz) vasomotion has also been proposed as a driving force for brain clearance and was previously experimentally shown to be associated with increased tracer movement in the CSF^5,11,28^. Unfortunately, the influence of resting-state vasomotion cannot be assessed using retrospective binning as was done with the cardiac and respiratory cycles, because it does not provide an accessible external triggering opportunity. Animal experiments have shown that low-frequency visual stimulation can be used to entrain vasodilation at the vasomotion frequency and thereby speed up clearance of fluorescently labeled tracers^5^. Moreover, in humans, it was recently shown that visual stimulation could drive global CSF flow in the fourth ventricle^25^. By using a similar stimulation strategy to entrain vasomotion, we showed that CSF mobility within the visual cortex area is significantly enhanced in humans during 0.1-Hz frequency visual stimulation and that the amplitude of this effect approaches the strength of cardiac and respiratory fluctuations. The ability of CSF-STREAM to measure CSF mobility within the stimulated region, located much closer to the production sites of metabolic ‘waste’ products, should be considered an important advantage over techniques that measure CSF flow at a single location at the exit of the brain, such as the aqueduct or the fourth ventricle.

The observed increase in CSF mobility by entraining vasomotion might mirror similar, albeit stronger, physiological fluctuations that occur during sleep. Indeed, during sleep, low-frequency oscillations in cerebral blood volume, measured using BOLD functional MRI (fMRI)^26,32^, are of similar amplitude as the ones measured in our study during a 0.1-Hz visual stimulation (Fig. 7c). As these oscillations are brain-wide during sleep, and not localized to a stimulated region, this could in turn lead to brain-wide enhancement of CSF mobility during sleep.

While the increase in CSF-mediated clearance during sleep is proposed to be a key feature of its proper function, clearance failure has been suggested to be part of the pathogenesis of CAA, a common small-vessel disease and contributor to dementia, which is characterized by vascular amyloid-β accumulation and subsequent brain lesions, such as hemorrhages. Our pilot study in patients with CAA provides in vivo confirmation of observations made in rodent models of CAA. We observed regional alterations in CSF mobility and FA in patients with sporadic CAA compared to healthy controls, even in our relatively small sample: CSF mobility was increased by 20% and FA decreased by 10% within the SAS surrounding the MCA, whereas these parameters remained unchanged in PVS. Our findings are in line with the observations in the rodent model of CAA, where a similar 20% increase in CSF speed was found in the SAS at the skull base, whereas CSF speed in tissue remained unchanged^19^. The higher CSF mobility together with lower FA might indicate higher but more disorganized CSF mobility patterns in the SAS, with the CSF bypassing the tissue compartment. Such bypassing might be caused directly by amyloid-β accumulation in cortical or leptomeningeal vessels or indirectly by stiffening of arterioles and loss of smooth muscle cells^33^. Altogether, this could lead to decreased solute clearance from the brain tissue and thereby explain the counterintuitive finding of increased mobility in CAA: when the brain tissue stiffens or is waste-laden, CSF penetration into the brain tissue along perivascular channels will be more difficult, effectively enhancing CSF flow at the brain surface. Importantly, these findings also underscore the current gaps in our understanding of CSF mobility patterns and their modulation across neurological conditions, and warrants further investigations. Interestingly, we found that the distance of the CSF volume to the vessel is of importance in the SAS surrounding the MCA (Extended Data Fig. 6): when measuring in CSF located within ~1.7 mm from the MCA, CSF mobility and FA were different between patients with CAA and healthy controls, whereas when including CSF located further away from the MCA, the changes between groups attenuated. This might be explained by an additional membrane in the SAS along the major cerebral arteries that compartmentalizes the SAS, as recently proposed by Eide and Ringstad^34^.

CSF-STREAM was inspired by the long echo-time diffusion tensor imaging (DTI) approach proposed by Harrison and colleagues to measure CSF mobility in the SAS surrounding the MCA in the rat brain^35^. Isolation of the CSF signal is essential to the successful measurement of CSF mobility in PVS because it ensures that the measured signal solely originates from the CSF and not from slow-flowing blood in the vessel located inside the PVS. A good separation of the CSF signal was achieved, as shown in Fig. 1, Extended Data Fig. 1 and Supplementary Video 1. The use of motion-sensitizing gradients applied during T_2_ preparation instead of a traditional diffusion sequence enabled the robust use of a multi-shot turbo spin-echo (TSE) readout for which image quality and achievable resolution are substantially higher than with an echo planar imaging (EPI) readout that is usually the backbone of DTI scans: the motion information encoded in the T_2_ preparation is stored in the longitudinal plane and therefore avoids phase accruals that would make the multi-shot reconstruction subject to image artifacts. Altogether, using the proposed CSF-STREAM technique, we showed that CSF mobility could be measured noninvasively and at a high spatial resolution in healthy individuals and patients by combining accelerated ultra-high-field MRI while exploiting the magnetic properties of the CSF and motion-sensitizing gradients.

The measured CSF mobility values are more than ten times higher than water diffusion values, even in PVS (~1 × 10^−3^mm^2^ s^−1^ for water diffusion in brain tissue^36^ versus 10–50 × 10^−3^ mm^2^ s^−1^ for CSF mobility). This high CSF mobility combined with a high FA indicates that the physiological process behind CSF mobility is not pure diffusion or plug flow, but more probably laminar flow or back-and-forth motion^22^. This is further substantiated by the influence of physiology on CSF mobility: if the measured CSF mobility was diffusion-dominant, then no effect of the cardiac or respiratory cycles should have been observed^22,23,27^. The motion sensitizing used in this study was 3.5 mm s^−1^, which was sufficient to partially attenuate the CSF signal in PVS, indicating that the measured CSF mobility values probably correspond to velocities in the order of magnitude of ~1–2 mm s^−1^ (Supplementary Figs. 1–3). As the current sequence does not allow for measuring the phase accrual generated by the motion-sensitizing gradients, it is not possible to determine whether the CSF exhibits net flow in the PVS or if CSF mobility in PVS would be better described as a mixing, oscillatory phenomenon: the direction (toward one end of the axis of movement or the other; for example, right to left or left to right) cannot be determined with the current sequence. Still, a potential propagation pattern of CSF mobility across cardiac phases can be observed (Figs. 3 and 4 and Supplementary Videos 2 and 3), suggesting that CSF mobility varies in distinct spatially coherent waves driven by the cardiac and respiratory cycles. This could further enhance the efflux of waste products from the neuropil to the SAS.

A further limitation of the current approach is the long scan duration that leads to a higher sensitivity to motion (two datasets had to be excluded because of motion artifacts) and to a low intrinsic temporal resolution, which limits the study of pulsatile dynamics that cannot be predicted in advance or using an external trigger (for example, vasomotion as opposed to cardiac or respiratory dynamics). Regarding the scan duration, the current *k-*space sampling rendered a sufficient signal-to-noise ratio, allowing to further split the *k*-space to study the effect of cardiorespiratory pulsations. This suggests that for baseline CSF mobility measurements (where retrospective binning is not needed), further acceleration can be achieved. Shorter acquisition times resulting from such higher acceleration combined with the use of prospective motion correction, for example, by navigators^37,38^, would also help reduce sensitivity to motion. However, even with the current scan times, it was possible to identify changes in CSF mobility in patients with a neurodegenerative disease.

The truly noninvasive nature of the developed technique, in contrast to techniques using tracer injections, allows the inclusion of this methodology into longitudinal, patient and population studies. It also makes the sequence repeatable (that is, applicable several times in a row), which would be essential when applied to sleep research or to monitor disease progression, for example, in neurodegenerative diseases. The information obtained from such studies could lead to important insights into the ways human brain pathologies are affected by impaired brain clearance and lead to new ways of improving brain health.

## Conclusion

CSF-STREAM enables detailed measurement of CSF mobility in humans, from large CSF-filled spaces down to PVS surrounding penetrating vessels in a fully noninvasive manner. Cardiac and respiratory fluctuations induced comparable oscillations in PVS, whereas in larger CSF spaces at the base of the brain, the cardiac cycle was the main driving force of CSF mobility. Moreover, regional alterations in CSF mobility were found in patients with a presumed brain clearance disorder. Finally, CSF mobility in the visual cortex could be enhanced through entraining vasomotion at 0.1 Hz.

## Methods

### Participants

#### Healthy, younger cohort

A total of 24 healthy individuals (aged 33 ± 13 years, 20 females, 4 males) were scanned: 14 individuals (35 ± 15 years, 11 females, 3 males) were enrolled in the first study to evaluate fluctuations in CSF mobility across cardiac, respiratory and random phases. One individual (female, 26 years old) was excluded because of motion artifacts and two because of insufficient quality of the cardiac signal (both female, 20 and 60 years old). Motion was identified in the reconstructed images as blurring of the images and duplication of brain structures. Ten (30 ± 9 years old, 9 females, 1 male) participated in the second study to investigate the effect of a visual stimulation on CSF mobility, of which one (female, 18 years old) was excluded because of motion artifacts. Six individuals (28 ± 2 years old, 5 females, 1 male) participated in both studies. All were screened for MRI contraindications and provided written informed consent. All experiments were performed in accordance with the Leiden University Medical Center Institutional Review Board under authorization no. P07.096. Each participant of this cohort was compensated with a €20 voucher for every scan session they completed.

#### Cohort with CAA

Patients with CAA (*n* = 8, 72 ± 7 years old, 1 female, 7 males) were recruited through the neurovascular outpatient clinic at University Hospital Bonn. The diagnosis of probable CAA was independently confirmed by a board-certified neuroradiologist according to the Boston Criteria v.2.0 (ref. ^29^). Age-matched and sex-matched healthy participants (*n* = 8, 73 ± 7 years old, 2 females, 6 males) were recruited through the DANCER cohort, a neurologically unaffected control cohort of the German Center for Neurodegenerative Diseases. The study was approved by the Ethics Committee of University Hospital Bonn. Written informed consent was obtained for each participant. The study participants of the cohort with CAA were compensated with €100 and provided complimentary parking.

Medical history was obtained from each participant to record relevant previous diseases, cardiovascular risk factors, degree of disability, lifestyle factors and physical activity. The degree of disability was assessed using the modified Rankin Scale. Physical activity was assessed using the Physical Activity Scale for the Elderly. Global cognitive status was assessed using the Montreal Cognitive Assessment test. Trail Making Test parts A and B were used to assess executive function and processing speed. Symptoms of depression were assessed using the Center for Epidemiologic Depression Scale, the revised Becker Depression Inventory and the Geriatric Depression Scale.

### MRI scans acquisition

#### Healthy, younger cohort

All scans were acquired using a 7 Tesla MRI scanner (Achieva, Philips, release 7TR5C-CDAS-BUCKEYE2-SWID171) equipped with a quadrature birdcage head coil and a 32-channel receive coil array (Nova Medical).

##### Anatomical three-dimensional T_1_ scan

Three-dimensional (3D) T_1_-weighted images were acquired using the following parameters: field of view (FOV) = 246 × 246 × 225 mm^3^, flip angle = 7°, TE = 1.9 ms, repetition time (TR) = 4.2 s, spatial resolution = 0.9 mm isotropic and acquisition time = 142 s.

##### CSF-STREAM

High-resolution, whole-brain (0.45 mm isotropic voxel size, FOV = 250 × 250 × 190 mm; 3D images were acquired with a TSE sequence: TE = 495 ms, TR = 3.4 s, TSE factor = 146, excitation and refocusing flip angle = 90°). This long TE readout was combined with a T_2_ preparation module (duration = 37 ms, two refocusing pulses) to allow the insertion of motion-sensitizing gradients and to further isolate the CSF signal.

To accelerate acquisition, *k*-space undersampling in the *ky* and *kz* phase encoding directions compatible with compressed sensing reconstruction was performed using the Amsterdam UMC PROUD patch^39^, based on a pseudoradial variable density (density decay = 0.5) sampling pattern with a fully sampled 29 × 29 autocalibration area in the center of the *k*-space. This way, an acceleration factor of 17 was achieved, which allowed to obtain high-spatial-resolution, whole-brain, static CSF images in 5 min and 30 s. Subsequently, motion-sensitizing gradients were included in the T_2_ preparation to encode CSF mobility. Seven sets of volumes (subscans) were acquired: one without motion-sensitizing gradients and six with gradients applied in different orthogonal directions. In practice, motion-sensitized gradients of 5 mm s^−1^ were played out on two axes simultaneously, resulting in a diagonal direction with a motion-encoding of 5/√2 = 3.5 mm s^−1^. The acquisition time per subscan was 5 min and 30 s, yielding a total scan time of 38 min and 30 s.

##### Visual stimulation scout fMRI scan

A visual scout fMRI BOLD scan was acquired to locate the visual cortex. The acquisition parameters were as follows: FOV = 222 × 190 mm^2^, TE = 22 ms, TR = 2 s, 35 slices, voxel size: 1.97 × 1.74 mm^2^ in-plane, 2-mm slice thickness, EPI factor = 43, 60 repetitions (time points), acquisition time = 128 s, flip angle = 70°. The visual stimulus consisted of three blocks of an 8-Hz flashing radial black-and-white checkerboard pattern for 20 s alternated with 20 s of a fixed gray screen as rest condition.

Using the aforementioned scans, two studies were performed in the healthy, younger cohort.

##### Study 1: CSF mobility across cardiac, respiratory and random phases

This protocol consisted of an anatomical 3D T_1_ scan, a CSF-STREAM scan (one scan = one non-motion-sensitized subscan + 6 motion-sensitized subscans) and a visual stimulation scout scan (except for the first four individuals). During acquisition, the heart rate was continuously recorded using a peripheral pulse unit and the respiratory rate using a belt wrapped around the individual’s chest; both physiological monitoring devices were the standard equipment as supplied by the vendor (Philips).

##### Study 2: entrained vasomotion using a 0.1-Hz visual stimulation

This protocol consisted of an anatomical 3D T_1_ scan, a CSF-STREAM scan and a visual stimulation scout scan. During each of the seven CSF-STREAM subscans, a visual stimulus was shown during the first half of the subscan, and a gray screen during the second half of the subscan. The stimulation paradigm consisted of an 8-Hz flashing radial black-and-white checkerboard pattern for 5 s alternated with 5 s of a fixed gray screen, altogether leading to a 0.1-Hz stimulation frequency. To sample the *k*-space homogeneously in both subscan halves, *k*-space sampling was readjusted by first acquiring the odd TSE shots of the original *k*-space sampling in the first half and subsequently the even TSE shots during the second half.

#### Cohort with CAA

MRI data were acquired with a 7 Tesla MRI system (Siemens Healthineers) using a head array coil with 32 receive and eight transmit channels (Nova Medical). Scans were performed in the morning between 9:30 and 11:30.

The scan protocol included a T_1_-weighted multi-echo magnetization-prepared rapid gradient echo (0.80 mm isotropic, TR = 2,800 s, TI = 1,100 ms, scan time = 4 min) and a CSF-STREAM scan (0.50 mm isotropic, TE = 515 ms, TR = 3.400 s, TSE factor = 146, refocusing flip angle = 70°, 12× Poisson disk undersampling scheme, acquisition time = 4 min and 15 s per subscan, motion-sensitizing gradients of 4 mm s^−1^). In practice, like the study in healthy, young volunteers, motion-sensitized gradients of 5.6 mm s^−1^ were played out on two axes simultaneously, resulting in a diagonal direction with a motion-encoding of 4 mm s^−1^. Additionally, susceptibility-weighted images (0.50 mm isotropic) were acquired to quantify cerebral microbleeds and to identify superficial siderosis.

### CSF-STREAM image reconstruction

#### Healthy, younger cohort

All CSF-STREAM reconstructions of this cohort were performed offline in MATLAB 2018b (MathWorks), using an in-house-built reconstruction pipeline developed within ReconFrame (v.4.3.1, GyroTools) in combination with the open-source Berkeley Advanced Reconstruction Toolbox (BART)^40^ v.0.4.03.

##### Study 1: retrospective cardiac, respiratory and random binning

After acquisition, each of the seven CSF-STREAM subscans was reconstructed three times: the *k*-space profiles were retrospectively binned in six phases, using retrospective binning to either (1) the cardiac cycle, (2) the respiratory cycle or (3) random phases (negative control). The R-R cardiac peaks and the respiration peaks were detected automatically from the recorded cardiac and respiratory traces using a MATLAB script (using the findpeaks function); the results were checked manually for each scan and corrected if wrongly detected. Random phases were generated using the randi MATLAB function. The retrospective binning in six phases was performed in two steps to preserve image quality: the *k*-space was binned twice in three phases, with a one-sixth phase shift between the two steps, and subsequently combined in one dataset (Extended Data Fig. 3). The T_2_ preparation preceding each TSE shot was taken as reference and the signal in the subsequent TSE shot was considered to be dependent on this specific cardiac, respiratory or random phase^41,42^. Each reconstruction step was performed using BART’s pics command with total variation in the temporal domain, with a regularization factor of 0.005 and input coil sensitivities estimated from the *k*-space center using BART’s caldir command. Altogether, this generated six tensors per driving force per individual (that is, one tensor per cardiac, respiratory, and random phases).

##### Study 2: 0.1-Hz visual stimulation

The two half *k*-spaces (0.1-Hz stimulation versus rest) of each of the seven CSF-STREAM subscans were reconstructed using BART’s pics command with total variation in the temporal domain, with a regularization factor of 0.005 and input coil sensitivities estimated from the *k*-space center using BART’s caldir command. Per individual, this resulted in a set of two tensors: one with 0.1-Hz stimulation and one at rest.

#### Cohort with CAA

Image reconstruction of the CSF-STREAM subscans was achieved using the pics command from the BART Toolbox with L1 regularization (regularization factor = 0.002) and 30 iterations. Input coil sensitivities were estimated from a fully sampled gradient echo pre-scan using BART’s ecalib command.

### Postprocessing: healthy, younger cohort

#### CSF mobility, FA and principal orientation of CSF mobility

Intra-individual images were registered using elastix v.4.9.0 (ref. ^43^). CSF mobility, its principal orientation and FA were modeled using a MATLAB script^44^ by computing the mean eigenvalue of a rank-two positive definite tensor, analogous to DTI. CSF mobility is a measure of the amount of movement that the CSF undergoes within a given time in a voxel as a function of intra-voxel dephasing of the signal due to the application of bipolar gradients. It is measured in mm^2^ s^−1^ and is calculated in a similar way as an apparent diffusion coefficient.

For the first study that investigated the effect of the cardiac, respiratory and random pulsations, six CSF mobility and FA maps were created (one for each phase) per driving force per individual. Change in CSF mobility and change in FA maps were obtained for each cardiac, respiratory and random phase after normalizing the maps voxel-wise to the mean value over phases.

Changes in CSF mobility and FA across driving forces were investigated in six ROIs. As shown in Figs. 3 and 4, the fluctuations across phases varied spatially, such that the phase of maximum signal was location-dependent. To avoid phase cancellation within an ROI and hence potential smoothing of the effect of a driving force, the time profiles were realigned voxel-wise before calculating the average CSF mobility and FA changes in an ROI. That way, phase 1 always contained the maximum signal change and was discarded from the plots in Fig. 5 and Extended Data Fig. 4.

To quantitatively compare how CSF mobility varied across the proposed driving forces, the original change in CSF mobility over six phases (that is, without the realignment mentioned in the previous paragraph) was fitted voxel-wise to a sine function. The fit quality, maximum amplitude and phase of the maximum amplitude were the fitted parameters. The amplitude of voxels with low-fit quality (*R*^2^ < 0.5) was set to 0 to better represent the absence of coherent change across phases. For example, in the right panel of Extended Data Fig. 5b, the fitted curve should ideally be flat (that is, with an amplitude of 0% instead of 12%) because the signal pattern is noisier (‘zig-zag’) than coherent across phases.

For the second study investigating the effect of entrained vasomotion on CSF mobility, two CSF mobility maps were created: one acquired during the 0.1-Hz visual stimulation and another at rest. The relative difference between the average CSF mobility during the 0.1-Hz stimulation and rest (no stimulation) was computed to investigate the effect of entrained vasomotion. In individuals who participated in both studies, the effect induced by entrained vasomotion was compared to that from cardiac, respiratory and random cycles (Fig. 7d) by computing the difference between the maximum and minimum change in CSF mobility over cardiac, respiratory and random phases.

All results were visualized using MATLAB and Paraview (v.5.6.0)^45^. When plotting CSF mobility and FA volume renderings or CSF mobility change maps, voxels where the CSF signal was lower than 150 were masked to exclude noise. The CSF mobility orientation plots were visualized in smaller volumes of interest. Before computing the CSF mobility orientation as described above, the resolution of the small volume of interest of the seven CSF-STREAM subscans was doubled using the MATLAB function imresize3 to better show the orientation in the selected regions.

#### fMRI processing

##### *Z*-score map generation

fMRI data processing was carried out using the FMRI Expert Analysis Tool, which is part of FSL, v.6.0. The following pre-statistics processing was applied: motion correction using MCFLIRT^46^; slice-timing correction using Fourier-space time-series phase-shifting; non-brain removal using the Brain Extraction Tool^47^; spatial smoothing using a Gaussian kernel of full width at half maximum of 3 mm; grand mean intensity normalization of the entire four-dimensional dataset by a single multiplicative factor; and high-pass temporal filtering (Gaussian-weighted least-squares straight line fitting, with *σ* = 50 s). To investigate the possible presence of unexpected artifacts or activation, independent component analysis-based exploratory data analysis was carried out using MELODIC^48^. The statistical analysis of the time series was carried out using FILM with local autocorrelation correction^49^.

##### *Z*-score template (study 1)

As the first four individuals of study 1 did not have a visual stimulation scout scan, a mean *z*-score template was generated to create a visual cortex mask for study 1, using the scout scan of a subset of ten individuals (from studies 1 and 2; only one scout scan per individual was included, meaning that scans from the six individuals included in both studies were only included once). To create this *z*-score template, the ten BOLD scans were registered to the 3D T_1_ scans using a boundary-based registration^50^. This transformation was applied to the *z*-score maps. Then, 3D T_1_ scans were registered to the Montreal Neurological Institute space using FSL’s FLIRT^46,51^ and FNIRT^52^. The resulting warpfield was applied to the registered *z*-score maps. The template was then generated using a one-sample group mean generalized linear model within FreeSurfer v.7.2 (ref. ^53^) and transformed into the CSF mobility space of each individual participant of study 1.

##### Individual *z*-score map (study 2)

For study 2, a visual stimulation scout scan was available for each individual. Therefore, for this study, individual *z*-score maps were used to best detect the visual cortex in each individual. Thus, the person-specific *z*-score maps were registered to each individual CSF mobility space in the following way. First, the BOLD scans were registered to the 3D T_1_ scans using a boundary-based registration^50^. Subsequently, the 3D T_1_ scans were skull-stripped and segmented into brain tissue types^54^. The obtained CSF probability map was registered to the CSF mobility space with an Euler registration using elastix^43^. The resulting transformations were then applied to the *z*-score maps.

The obtained *z*-score maps were used to detect the visual cortex.

The BOLD signal amplitude was calculated from the registered, motion-corrected, slice-time-corrected BOLD images. The three stimulation patterns (3 × 20 dynamics) were first averaged together, then the relative difference between the baseline signal (averaged over dynamics 5–10) and maximum signal (averaged over dynamics 15–20) was computed.

#### ROI definition

ROIs were manually delineated using anatomical landmarks on the non-motion-sensitized CSF scan (Fig. 2) using ITK-SNAP v.3.8.0 (ref. ^55^) as follows:

- The fourth ventricle ROI was drawn over 13 transversal slices.
- The ROI delimiting the SAS around the MCA was drawn over seven transversal slices on the left MCA branch.
- The motor cortex SAS sulci ROI was drawn over ten transversal slices.
- PVS in the BG were identified over sagittal slices as CSF-filled spaces around the lenticulostriate arteries.
- The ROI of PVS surrounding penetrating vessels in the white matter was drawn over 25 transversal slices in the CSO, starting from the slice directly above the lateral ventricles.
- The blood ROI was drawn inside one MCA branch over two slices.
- The noise ROI was drawn outside the brain, on the two central sagittal slices and on the corners of the same slice where the SAS-MCA ROI was drawn.

For study 1, the visual cortex ROI was defined based on the template *z*-score output (four datasets of study 1 did not contain a visual stimulation scout scan). A threshold *z*-score greater than 3.5 was used for all individuals of this study. Only the largest cluster of contiguous voxels was included in the mask. As the resolution of the fMRI scan used to create the *z*-score map was much lower than that of the CSF-STREAM, the obtained area not only contained the visual cortex but also the CSF in its vicinity.

To extract the final areas of interest, the manually delineated ROIs and the visual cortex ROI were multiplied with a CSF mask, thus including only voxels containing CSF and not noise. This CSF mask was created by first thresholding the non-motion-sensitized CSF scan using a threshold of 150 a.u. After visual inspection, this threshold could be adapted individually to assure proper selection of PVS. For study 1, if a voxel had a change in CSF mobility higher than 50% in one or more of the cardiac, respiratory and random datasets, it was excluded from the mask. Voxels that had no included neighboring voxels (‘lonely’ voxels) were also excluded from the mask. For the two PVS ROIs, an additional Frangi filter^56^ (0.6 < *σ* < 1 with a step of 0.2, Frangi vesselness constant = 0.5) was applied to ensure the exclusive inclusion of vessel-like structures and exclusion of noise.

For study 2, a visual stimulation scout scan was available for all individuals; therefore, we used individual *z*-score maps to create the visual cortex ROIs. A threshold *z*-score greater than 7 was used to define the visual area and a threshold *z*-score smaller than 1 was used to define a control region (rest of the brain). For the visual cortex ROI, only the largest cluster of contiguous voxels was included in the ROI. Next, to ensure only voxels containing CSF were included and not noise, a mask based on the non-motion-sensitized CSF scan was created using a threshold of 150 a.u. Voxels for which the change in CSF mobility between the two conditions (stimulation ON and OFF) was higher than 50% were considered as noise and excluded from the ROI. As the resolution of the fMRI scan used to create the *z*-score map was much lower than that of CSF-STREAM, the obtained area not only contained the visual cortex but also the CSF around the visual cortex (SAS and PVS).

The effect induced by the cardiac and respiratory cycles was compared to the changes induced by entrained vasomotion in individuals who participated in both studies. To that end, the personalized visual area mask created for study 2 was registered to the CSF space of study 1 using elastix.

### Postprocessing: cohort with CAA

#### CSF mobility and FA

The CSF-STREAM subscans were first interpolated from a 0.50-mm to a 0.17-mm isotropic resolution and co-registered using elastix. Subsequently, the mean eigenvalue of a rank-two positive definite tensor was computed (DTI postprocessing) using Python 3.10 to assess CSF mobility and FA. To minimize the effects of background noise, a cutoff value of 0.15 mm^2^ s^−1^ was used to exclude all voxels with unphysiologically high CSF mobility values.

#### ROI definition

##### SAS-MCA segmentations

The M1 segment of the MCA was first segmented semimanually using anatomical landmarks on the non-motion-sensitized CSF scan using MITK v.2022.10. MCA segmentation was then inflated using the imdilate MATLAB function to create a CSF mask containing the SAS around the MCA; inflation was done from 0.17 mm up to 3.00 mm with a step size of 0.17 mm. To ensure only the CSF signal was selected in the mask, voxels with a low CSF signal in the non-motion-sensitized scan were excluded.

##### PVS segmentations

Parcellated atlases from the T_1_ scan were generated using FreeSurfer v.6.0. White matter segmentations, derived from the FreeSurfer parcellation, were manually corrected if necessary. To capture comparable ROIs of the CSO in all participants, the eyes and optic chiasm served as anatomical landmarks for reference plane definition. The dimensions of the CSO segmentation encompassed the entire white matter above the lateral ventricles. PVS within the defined CSO segmentation were semiautomatically segmented using a Meijering filter-based approach^57^ with a global threshold on the interpolated non-motion-sensitized scan of CSF-STREAM (0.17 mm isotropic).

#### Assessment of microbleeds

Cerebral microbleeds were quantified and superficial siderosis was identified by a board-certified neuroradiologist with 8 years of experience according to the STRIVE-2 rating scale.

### Statistical analysis

Within each ROI, we evaluated whether the fit quality and amplitude of the change in CSF mobility were significantly different between driving forces. A post-hoc Bonferroni-corrected pairwise comparison between driving force effects was performed using a two-sided Wilcoxon signed-rank test when the Friedman test was significant. To evaluate the effect of the visual stimulation, a two-sided Wilcoxon signed-rank test was performed on the average CSF mobility values with and without stimulation from each individual. These aforementioned statistical analyses were performed in MATLAB.

To evaluate the evidence for a correlation between BOLD amplitude and change in CSF mobility in response to a visual stimulation, a Bayesian correlation analysis was performed using the JASP software^58^ (v.0.16.4). A stretched beta prior with a width of 1.0 was used; the Bayes factor was tested to be stable over a range of prior settings.

To evaluate differences in CSF mobility, FA, ROI volume and patient information between individuals with CAA and healthy controls, a two-sided Mann–Whitney *U*-tests was performed.

Unless mentioned otherwise, shaded error areas and error bars represent CIs of s.d. × 1.96 (√*n*)^−^^1^, *n* being the number of included individuals.

Box plots were plotted using the MATLAB boxplot function: in each box plot, the central line indicates the median; the bottom and top edges of the box indicate the 25th and 75th percentiles, respectively. The whiskers extend to the most extreme data points not considered outliers; outliers are plotted individually using the ‘+’ marker symbol.

Preliminary parts of this work were presented at conferences^41,42,59,60^.

### Reporting summary

Further information on research design is available in the Nature Portfolio Reporting Summary linked to this article.

## Online content

Any methods, additional references, Nature Portfolio reporting summaries, source data, extended data, supplementary information, acknowledgements, peer review information; details of author contributions and competing interests; and statements of data and code availability are available at 10.1038/s41593-025-02073-3.

## Supplementary information

Supplementary Information Supplementary information containing information on the motion encoding optimization and on the interpretation of mobility coefficients.

Reporting Summary

Supplementary Video 1 **High-resolution, whole-brain CSF signal**. Whole-brain CSF signal measured using the non-motion-sensitized reference scan, shown in one individual.

Supplementary Video 2 **Animation of CSF mobility change across driving forces in the subarachnoid space around the circle of Willis**. CSF mobility change from the mean value over phases (in %) across the cardiac (left), respiration (middle) and random (right) cycles in one representative individual (same data as in Fig. 3, but as a gif). Please note that a linear interpolation was applied between the phases to visually smoothen the video.

Supplementary Video 3 **Animation of CSF mobility change across driving forces in PVS around penetrating vessels**. CSF mobility change from the mean value over phases (in %) across the cardiac (left), respiration (middle) and random (right) cycles in one representative individual (same data as in Fig. 4, but as a gif). Please note that a linear interpolation was applied between the phases to visually smoothen the video.

Supplementary Data Statistical source data for Supplementary Fig. 1.

## Source data

Source Data Fig. 2 Statistical source data.

Source Data Fig. 3 Statistical source data.

Source Data Fig. 4 Statistical source data.

Source Data Fig. 5 Statistical source data.

Source Data Fig. 6 Statistical source data.

Source Data Fig. 7 Statistical source data.

Source Data Fig. 8 Statistical source data.

Source Data Extended Data Fig. 1 Statistical source data.

Source Data Extended Data Fig. 4 Statistical source data.

Source Data Extended Data Fig. 5 Statistical source data.

Source Data Extended Data Fig. 6 Statistical source data.

## Figures and Tables

**Extended Data Table 1:**
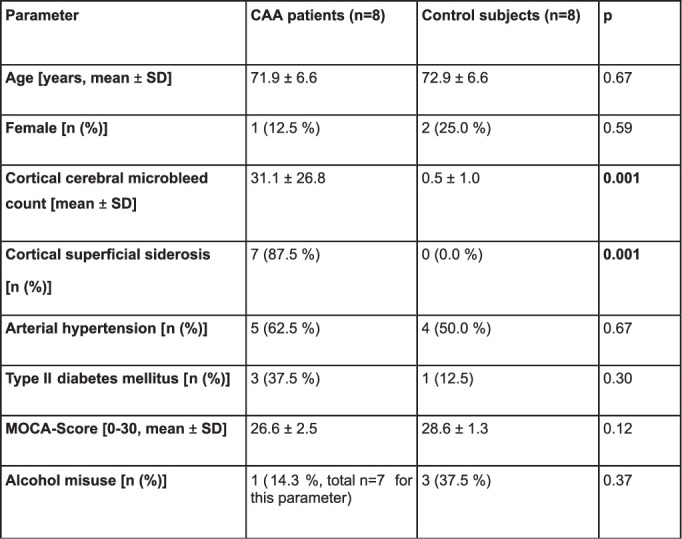
Participant characteristics of the CAA sub-study

Numbers indicate mean absolute values and standard deviations. CAA, cerebral amyloid angiopathy; For arterial hypertension diagnosis, participants were asked whether as a known hypertension diagnosis was present and the clinician checked if a medication-based hypertension therapy was performed; MOCA, Montreal cognitive assessment test (0-30 points, >25 points: no impairment, 18-25 points: at least mild cognitive impairment, 10-17 points: moderate cognitive impairment <10 points: severe cognitive impairment); alcohol misuse was defined as alcohol consumption of ≥16 drinks/week over a time period of at least one year. Groups were compared using two-sided Mann-Whitney U-tests.

## Data Availability

Source data are available via Zenodo at 10.5281/zenodo.15882390 (ref. ^61^). Source data are provided with this paper.

## Code availability

The code used to process the CSF-STREAM data is available on Zenodo at 10.5281/zenodo.15882390 (ref. ^61^; link to GitHub repository: https://github.com/lydianehirschler/CSF-STREAM).
