# Supplementary material for: Region-specific drivers of CSF mobility measured with MRI in humans
Source: Nat Neurosci. 2025 Oct 14;28(11):2392–401. doi: 10.1038/s41593-025-02073-3 (PMC12586159; doi:10.1038/s41593-025-02073-3)
Supplement: Supplementary file 1 — Supplementary information containing information on the motion encoding optimization and on the interpretation of mobility coefficients. [file 41593_2025_2073_MOESM1_ESM.pdf]

nature neuroscience

Supplementary information

https://doi.org/10.1038/s41593-025-02073-3

Region-specific drivers of CSF mobility measured with MRI in humans

In the format provided by the authors and unedited

Supplementary information

Amount of motion encoding – To optimally set the strength of the motion-sensitizing gradients, five CSF-STREAM sub-scans were acquired in three subjects: one sub-scan without motion-sensitizing gradients, and four sub-scans with different motion-sensitizing gradient strengths (2, 3.5, 5 and 7 mm/s). All other imaging parameters were the same as in the original CSF-STREAM sequence. Images were reconstructed using BART's *pics* command using a wavelet transform in the spatial dimensions with a regularization factor of 0.0015 and input coil sensitivities estimated from the k-space center using BART's *ecalib* command.

The signal attenuation due to motion encoding was calculated as the relative difference between a sub-scan with motion-sensitizing gradients and the sub-scan without motion-sensitizing gradients. The resulting attenuation was evaluated in two manually-drawn regions of interest: in the SAS around the MCA as well as in PVS of the basal ganglia.

A motion-sensitizing gradient strength of 3.5mm/s was found optimal, as it attenuates CSF in both SAS and PVS, but does not reach too high (>80%) attenuations, which would make the calculation of CSF-mobility not accurate in these regions.

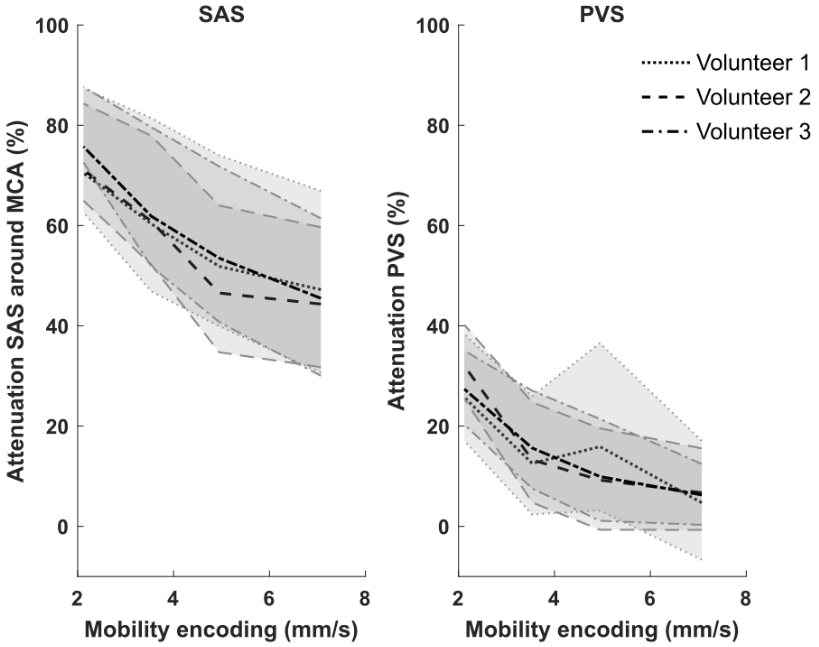

<u>Supplementary figure 1</u>: **Choice of the motion-sensitizing gradient strength evaluated in n=3 individuals**. Each black line represents the mean attenuation in one volunteer across motion-sensitizing gradient strengths. The shaded area displays the 30th and 70th percentiles over voxels in the region of interest (# of voxels: SAS-MCA: volunteer 1: 2623 voxels; volunteer 2: 1652 voxels; volunteer 3; 2560 voxels. PVS: volunteer 1: 824 voxels; volunteer 2: 667 voxels; volunteer 3: 548 voxels).

Interpretation of mobility coefficients – In the CSF-STREAM approach, CSF-mobility is calculated from the signal attenuation obtained using motion-sensitizing, as:

$$CSF_{mobility} = -\frac{1}{b}\ln(attenuation),$$

where b is the b-value calculated from the motion sensitizing gradients of the preparation module. In CSF-STREAM, a b-value of 13 s/mm^2 is applied in two directions, resulting in an effective b-value of 26 s/mm^2 in the diagonal direction. An alternative measure of motion encoding is the v_{enc}, which in CSF-STREAM is 5 mm/s applied in two directions, resulting in an effective v_{enc} of 5/√2 = 3.5 mm/s in the diagonal direction.

Attenuation of signal in CSF-STREAM can be the result of different flow scenarios (bulk flow, parabolic flow, oscillatory flow, random motion and combinations of these). We illustrate these different situations below:

BULK FLOW - If the motion in the voxel is bulk flow, the magnetization in that voxel accumulates a net phase φ that is proportional to the velocity v (same as in standard phase contrast imaging):

$$\varphi = \pi \times \frac{v}{v_{enc}}$$

with v_{enc} the velocity encoding value.

Due to the phase of the flip-up pulse, this phase accumulation leads to signal attenuation in the motion-sensitized image, which is therefore also directly related to velocity:

$$attenuation = \cos(\varphi)$$

$$attenuation = \cos\left(\pi \times \frac{v}{v_{enc}}\right)$$

By combining the previous equations, the conversion between CSF-mobility and velocity can be computed as:

$$CSF_{mobility} = -\frac{1}{b}\ln\left[cos\left(\pi \times \frac{v}{v_{enc}}\right)\right]$$

In this case, a CSF-mobility of 0.04 mm^2/s would be equivalent to a ~1.4 mm/s bulk flow velocity.

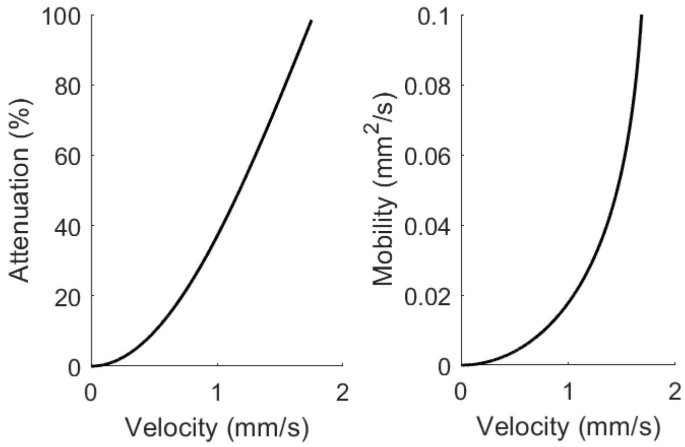

<u>Supplementary figure 2</u>: Signal attenuation (left) and corresponding mobility (right) calculated for different bulk flow velocities.

<u>PARABOLIC FLOW</u>: If the flow profile within the voxel is parabolic, then the signal can be integrated over the velocity profile as:

$$attenuation = \int_0^R 2\pi r \cos\left(\frac{\pi}{v_{enc}}\, v_{max}\left(1 - \frac{r^2}{R^2}\right)\right) dr$$

$$= \text{sinc}\left(\pi \times \frac{v_{max}}{v_{enc}}\right)$$

with R the radius of the tube and v_{max} the maximal flow velocity. The CSF-mobility measured in that case would be as follows:

$$CSF_{mobility} = -\frac{1}{b}\ln\left[\text{sinc}\left(\pi \times \frac{v_{max}}{v_{enc}}\right)\right],$$

Therefore, in the case of a parabolic flow profile, the maximum flow velocity equivalent to a 0.04 mm^2/s CSF-mobility would be ~2.5 mm/s.

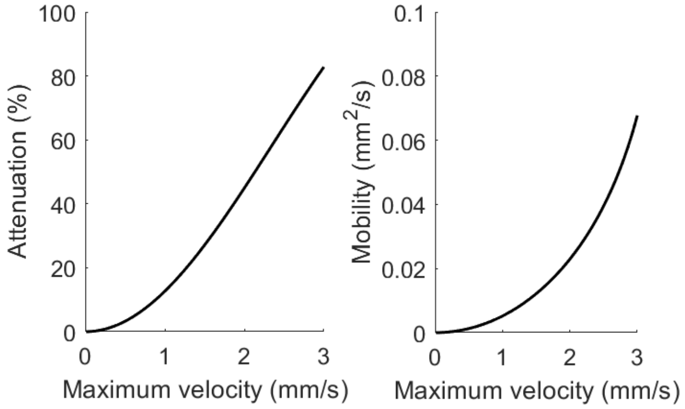

<u>Supplementary figure 3</u>: Signal attenuation (left) and corresponding mobility (right) calculated for parabolic flow profiles with varying maximum velocities.

RANDOM MOTION: Random motion can be modelled similar to diffusion and relies therefore completely on an apparent diffusion coefficient, similar as what Le Bihan et al have proposed for intra-voxel incoherent motion (IVIM), which models the attenuation of diffusion-weighted signal for an ensemble of randomly oriented capillaries of length *l* and with a blood velocity of *v*. Within the IVIM-concept, the apparent diffusion coefficient can then be shown to be equal to l*v/6. In a similar manner, the attenuation in CSF-STREAM will depend on the characteristic length and CSF-velocity. The subarachnoid trabeculae have been hypothesized to cause semi-random CSF flow patterns[1]. It is, however, difficult to assume a characteristic length for such motion patterns with possible values between 0.5 and approximately 100 μm^2. Average values of D* in the SAS were measured to be around 0.017 mm^2/s,[1] which is in line with our observations.

OSCILLATORY FLOW: Calculations of oscillatory flow are more challenging as they depend crucially on the frequency and amplitude of the oscillations. *Slow* oscillations will effectively be modelled as averaging over discrete flow conditions (see text above). These can therefore be obtained by averaging over the bulk or parabolic flow conditions. Conversely, in presence of *fast* oscillations, which occur on the order of time of the T_2-preparation, i.e. within 37 ms, the situation will evolve into the random motion condition.

Reference:

1. Rane Levendovszky, S., et al (2024). Preliminary investigations into human neurofluid transport using multiple novel non-contrast MRI methods. *Journal of Cerebral Blood Flow and Metabolism*. https://doi.org/10.1177/0271678X241264407

CAPTIONS OF SUPPLEMENTARY VIDEOS:

Supplementary video 1: High-resolution, whole brain CSF-signal. Whole brain CSF-signal measured using the non-motion-sensitized reference scan, shown in one individual.

Supplementary video 2: Animation of CSF-mobility change across driving forces in the subarachnoid space around the circle of Willis. CSF-mobility change from the mean value over phases (in %) across the cardiac (left), respiration (middle) and random (right) cycles in one representative individual (same data as in Fig. 3, but as a gif). Please note that a a linear interpolation was applied between the phases to visually smoothen the video.

Supplementary video 3: Animation of CSF-mobility change across driving forces in PVS around penetrating arteries. CSF-mobility change from the mean value over phases (in %) across the cardiac (left), respiration (middle) and random (right) cycles in one representative individual (same data as in Fig. 4, but as a gif). Please note that a a linear interpolation was applied between the phases to visually smoothen the video.
