# Supplementary material for: Region-specific drivers of CSF mobility measured with MRI in humans
Source: Nat Neurosci. 2025 Oct 14;28(11):2392–401. doi: 10.1038/s41593-025-02073-3 (PMC12586159; doi:10.1038/s41593-025-02073-3)
Supplement: Supplementary file 2 — Reporting Summary [file 41593_2025_2073_MOESM2_ESM.pdf]

nature portfolio

Corresponding author(s): Lydiane Hirschler

Last updated by author(s): Jul 15, 2025

Reporting Summary

Nature Portfolio wishes to improve the reproducibility of the work that we publish. This form provides structure for consistency and transparency in reporting. For further information on Nature Portfolio policies, see our Editorial Policies and the Editorial Policy Checklist.

Statistics

For all statistical analyses, confirm that the following items are present in the figure legend, table legend, main text, or Methods section.

n/a	Confirmed	
☐	☒	The exact sample size (n) for each experimental group/condition, given as a discrete number and unit of measurement
☐	☒	A statement on whether measurements were taken from distinct samples or whether the same sample was measured repeatedly
☐	☒	The statistical test(s) used AND whether they are one- or two-sided<br>*Only common tests should be described solely by name; describe more complex techniques in the Methods section.*
☒	☐	A description of all covariates tested
☐	☒	A description of any assumptions or corrections, such as tests of normality and adjustment for multiple comparisons
☐	☒	A full description of the statistical parameters including central tendency (e.g. means) or other basic estimates (e.g. regression coefficient) AND variation (e.g. standard deviation) or associated estimates of uncertainty (e.g. confidence intervals)
☐	☒	For null hypothesis testing, the test statistic (e.g. F, t, r) with confidence intervals, effect sizes, degrees of freedom and P value noted<br>*Give P values as exact values whenever suitable.*
☐	☒	For Bayesian analysis, information on the choice of priors and Markov chain Monte Carlo settings
☒	☐	For hierarchical and complex designs, identification of the appropriate level for tests and full reporting of outcomes
☒	☐	Estimates of effect sizes (e.g. Cohen's d, Pearson's r), indicating how they were calculated

Our web collection on statistics for biologists contains articles on many of the points above.

Software and code

Policy information about availability of computer code

Data collection	Healthy cohort: Philips 7T scanner with a pulse programming environment 7TR5C-CDAS-BUCKEYE2-SWID171 using a head array coil with 32 receive and 2 transmit channels (Nova Medical, Wilmington, MA, USA).<br>CAA cohort: 7 Tesla Siemens MRI system (Siemens Healthineers, Erlangen, Germany) using a head array coil with 32 receive and 8 transmit channels (Nova Medical, Wilmington, MA, USA).
Data analysis	Berkeley Advanced Reconstruction Toolbox (BART) version 0.4.03, ReconFrame version 4.3.1, MATLAB 2018b, Elastix 4.9.0, FSL 6.0, Freesurfer 6.0 and 7.2, Paraview 5.6.0, ITK-SNAP 3.8.0, Python 3.10 (scipy.stats module), MITK version v2022.10, itk-elastix v0.19, JASP Version 0.16.4. Codes used to process the data for this paper are available at: https://github.com/lydianehirschler/CSF-STREAM and on Zenodo (link to github).

For manuscripts utilizing custom algorithms or software that are central to the research but not yet described in published literature, software must be made available to editors and reviewers. We strongly encourage code deposition in a community repository (e.g. GitHub). See the Nature Portfolio guidelines for submitting code & software for further information.

Data

Policy information about availability of data

All manuscripts must include a data availability statement. This statement should provide the following information, where applicable:

- Accession codes, unique identifiers, or web links for publicly available datasets
- A description of any restrictions on data availability
- For clinical datasets or third party data, please ensure that the statement adheres to our policy

Source data is available on Zenodo.

Research involving human participants, their data, or biological material

Policy information about studies with human participants or human data. See also policy information about sex, gender (identity/presentation), and sexual orientation and race, ethnicity and racism.

Reporting on sex and gender	Healthy younger cohort: When registering to this study, the volunteers reported their sex. Based on ethical approval, no selection on sex or gender was performed. Therefore, our sample is skewed towards young Caucasian females. However, as this is a basic technical and physiology study, we think this is justifiable.<br><br>CAA cohort: Screening and inclusion of CAA patients in the study was based on the chronological order of their visits to our CAA outpatient clinic (DZNE Bonn). No selection was made based on sex or gender. For all CAA patients, a healthy age- and sex-matched control was identified from the DANCER cohort, a proven neurologically healthy study cohort of the DZNE. The cohort includes more men than women. It is known that men tend to have an earlier onset and a more severe course of CAA. This might lead to CAA being diagnosed more frequently and/or earlier in men, which may have contributed to this asymmetry. However, it was not analyzed whether this male-to-female predominance is also reflected in the overall CAA population of our CAA clinic.
Reporting on race, ethnicity, or other socially relevant groupings	Healthy younger cohort: Although not asked, we also expect our participants to be higher educated than the average Dutch population.<br><br>CAA cohort: the CAA and healthy control groups do not differ significantly from each other.
Population characteristics	Healthy younger cohort: A total of 24 healthy individuals (age: 33±13 years, 20 females, 4 males) were scanned: 14 individuals (35±15 years, 11 female, 3 male) were enrolled in the first study to evaluate CSF-mobility fluctuations across driving forces. One individual (female, 26 years old) was excluded because of motion artefacts and two because of insufficient quality of the cardiac signal (both female, 20 and 60 years old). Ten (30±9 years old, 9 female, 1 male) participated in the second study to investigate the effect of a visual stimulation on CSF-mobility, of which one (female, 18 years old) was excluded due to motion artefacts. Six individuals (28±2 years old, 5 female, 1 male) participated in both studies.<br><br>CAA cohort: A total of 18 study participants (10 CAA, 8 HC, 3/18 female, age 72.9 (SD: 6.6) years) were scanned, two CAA patients were excluded because the MRI study had to be discontinued and no complete CSF-STREAM data were available for analysis. This resulted in CSF-STREAM analysis of 16 study participants (8 CAA, 8 HC, 3/16 female, age 72.4 (SD 6.6) years). We allowed one additional female participant in the healthy group, as we could not recruit a male subject of the corresponding age. The group difference regarding sex is not significant.
Recruitment	Healthy volunteers between the age of 18 and 65 years were recruited through flyers and advertisements. Each participant of the younger, healthy cohort was compensated with a €20 voucher for every scan session they completed.<br>Although not asked, we also expect our participants to be higher educated than the average Dutch population. This is however not likely to impact our results.<br><br>CAA patients were recruited through the neurovascular outpatient clinic at University Hospital Bonn. The diagnosis of probable CAA according to the modified Boston Criteria was independently confirmed by a board-certified neuroradiologist. Age- and sex-matched healthy participants were recruited through the DANCER cohort, i.e. a neurologically healthy control cohort of the DZNE. The study participants of this cohort were compensated with €100 and complimentary parking. Participant characteristics do not differ significantly from each other between the CAA and control groups; likelihood of self-selection bias is therefore low in this cohort.
Ethics oversight	Healthy younger cohort: Leiden University Medical Center Institutional Review Board: "Medisch-ethische toetsingscommissie Leiden Den Haag Delft (METC LDD)". (approval number P07.096).<br><br>CAA cohort: IRB of University Hospital Bonn Ethical (approval number 473/21).<br><br>Written informed consent was obtained for all participants in both cohorts.

Note that full information on the approval of the study protocol must also be provided in the manuscript.

Field-specific reporting

Please select the one below that is the best fit for your research. If you are not sure, read the appropriate sections before making your selection.

☒ Life sciences ☐ Behavioural & social sciences ☐ Ecological, evolutionary & environmental sciences

For a reference copy of the document with all sections, see nature.com/documents/nr-reporting-summary-flat.pdf

Life sciences study design

All studies must disclose on these points even when the disclosure is negative.

Sample size	Healthy younger cohort: Measurements were performed in a total of 20 healthy, younger subjects to show technological and biological variance.<br>CAA cohort: the final cohort consisted of 16 study participants (8CAA, 8HC).<br><br>As all the performed measurements were novel, no sample size calculation could be performed in advance, as there were no SD values available.<br><br>For the healthy, younger cohort, we included ~10 participants per sub-study, which is commonly done in MRI-sequence development that assesses physiology (cf Fultz et al Science 2019, Roefs et al Fluid and Barriers of the CNS 2024). We did find region-specific changes with driving forces in this number of participants, also indicating the sample size was adequate.<br><br>For the CAA study, we conducted a pilot/exploratory study to evaluate whether large brain clearance changes occur in CAA patients and to generate hypotheses for future confirmatory research. Furthermore, as no prior data existed on the effect size of CSF-mobility changes in humans with CAA, accurate power calculations were not possible at the start of the study. Our results suggest a relatively large effect size of CAA pathology (cohen's d=1.46).
Data exclusions	Healthy younger cohort: Two individuals were excluded because of motion artifacts and two because of insufficient quality of the cardiac signal.<br>CAA cohort: Two CAA patients were excluded because their MRI study had to be discontinued, and no complete CSF-STREAM data were available for analysis.
Replication	Measurements were performed in 20 subjects to show technological and biological variance. All attempts at replication were successful.
Randomization	For the healthy, younger cohort, participants were randomly assigned to experiments in a consecutive order depending on their availability on the scanning day.<br>For the CAA study, the allocation in CAA/control group was based on clinical diagnosis, as described in the methods.
Blinding	The reader who performed the region-of-interest (ROI) definition of the Middle Cerebral Artery (MCA) was blinded to the group assignment; however, it cannot be ruled out that conclusions regarding the presence of CAA could have been drawn from the imaging data (e.g., through larger perivascular space (PVS) volumes, although this is not an entirely reliable criterion). This could not be prevented; however, the reader did not have access to the CSF-mobility nor FA maps during ROI placement (the MCA ROI placement was performed on the non-motion sensitized image, possibly with the assistance of the T1-weighting), so the reader had no information on how the ROI placement would affect the CSF-mobility or FA values. The person performing statistical analysis was blinded to the group assignment until the final significance test. The segmentation of the PVS was performed semi-automatically. If necessary, the reader, who already performed MCA segmentation, corrected the semiautomatically calculated PVS segmentation masks for false positives on the non-motion sensitized image and T1w without access to the CSF-mobility map.

Reporting for specific materials, systems and methods

We require information from authors about some types of materials, experimental systems and methods used in many studies. Here, indicate whether each material, system or method listed is relevant to your study. If you are not sure if a list item applies to your research, read the appropriate section before selecting a response.

Materials & experimental systems

n/a	Involved in the study	
☒	☐	Antibodies
☒	☐	Eukaryotic cell lines
☒	☐	Palaeontology and archaeology
☒	☐	Animals and other organisms
☐	☒	Clinical data
☒	☐	Dual use research of concern
☒	☐	Plants

Methods

n/a	Involved in the study	
☒	☐	ChIP-seq
☒	☐	Flow cytometry
☐	☒	MRI-based neuroimaging

Clinical data

Policy information about clinical studies
All manuscripts should comply with the ICMJE guidelines for publication of clinical research and a completed CONSORT checklist must be included with all submissions.

Clinical trial registration	After consulting with the ethics committee of the University Hospital Bonn, there is no obligation to register the study in a clinical trial registry, as it is an observational study that does not involve any intervention and does not investigate any therapy outcome.
Study protocol	The study protocol is not publicly accessible (see above).
Data collection	Data collection was performed between August 2022 and April 2023.
Outcomes	The primary endpoints were chosen as CSF mobility and fractional anisotropy around the middle cerebral artery and in the perivascular spaces in the centrum semiovale of CAA patients compared to neurologically healthy patients. Secondary endpoints included the comparison of the PVS volume in the centrum semiovale between the two groups.

Plants

Seed stocks	na
Novel plant genotypes	na
Authentication	na

Magnetic resonance imaging

Experimental design

Design type	A new MRI-sequence (CSF-STREAM) is presented in this paper to measure CSF-mobility in humans both at resting state, during a visual stimulation and in presence of CAA.
Design specifications	- Scout scan fMRI scan to locate the visual cortex: The visual stimulus consisted of 3 blocks of an 8 Hz flashing radial black-and-white checkerboard pattern for 20 seconds alternated with 20 seconds of a fixed grey screen as rest condition.<br>- 0.1Hz visual stimulation during CSF-STREAM: 8 Hz flashing radial black and white checkerboard pattern for 5 s alternated with 5 s of a fixed grey screen, altogether leading to a 0.1 Hz stimulation frequency
Behavioral performance measures	none

Acquisition

Imaging type(s)	anatomical 3D T1 weighted structural scan, CSF-STREAM, scout fMRI scan
Field strength	7 Tesla
Sequence & imaging parameters	3D T1-weighted images: field-of-view = 246×246×225 mm3, flip angle = 7◦, echo time (TE) = 1.9 ms, repetition time (TR) = 4.2 s, spatial resolution = 0.9 mm isotropic, and acquisition time = 142 s.<br>- CSF-STREAM: 0.45 mm isotropic voxel-size, field-of-view = 250×250×190 mm, 3D turbo-spin echo readout (TSE), TE = 495 ms, TR = 3.4 s, TSE-factor 146, excitation & refocusing FA = 90◦. To accelerate the acquisition, pseudo-random undersampling was performed (compressed sensing) in the phase-encoded directions (ky and kz), using the Amsterdam UMC PROUD patch. For more information about CSF-STREAM, see methods.<br>- visual stimulation scout fMRI scan: field-of-view = 222×190 mm2, flip angle = 70◦, TE = 22 ms, TR = 2 s, 35 slices, 1.97×1.74 mm2 in-plane resolution, 2 mm slice thickness, EPI-factor = 43, 60 dynamics, acquisition time = 128 s.
Area of acquisition	whole brain imaging

Diffusion MRI ☐ Used ☒ Not used

Preprocessing

Preprocessing software	Analysis of fMRI scout scan: fMRI data processing was carried out using FEAT (FMRI Expert Analysis Tool) Version 6.00, part of FSL. The following pre-statistics processing were applied: motion correction using MCFLIRT; slice-timing correction using

Fourier-space time-series phase-shifting; non-brain removal using the Brain Extraction Tool (BET); spatial smoothing using a Gaussian kernel of FWHM 3 mm; grand-mean intensity normalization of the entire 4D dataset by a single multiplicative factor; high-pass temporal filtering (Gaussian-weighted least-squares straight line fitting, with sigma = 50s).

Normalization

Normalization for study 1: BOLD scans were registered to the 3D-T1 scans using a boundary based registration. This transformation was applied to the z-score maps. Then, 3D-T1 scans were registered to MNI-space using FSL's FLIRT and FNIRT. The resulting warpfield was applied to the registered z-score maps. The template was then generated using a one-sample group mean generalized linear model within FreeSurfer and transformed into the CSF-mobility space of each individual subject of study 1.
For study 2, a visual stimulation scout scan was available for each subject. Therefore, for this study, individual z-score maps were used to best detect the visual cortex in each subject.

Normalization template

MNI space

Noise and artifact removal

For fMRI scout scan: To investigate the possible presence of unexpected artefacts or activation, ICA-based exploratory data analysis was carried out using MELODIC. The statistical analysis of the time-series was carried out using FILM with local autocorrelation correction.

Volume censoring

n.a.

Statistical modeling & inference

Model type and settings

n.a.

Effect(s) tested

A Wilcoxon signed rank test was performed on the average CSF-mobility values with and without stimulation from each subject to evaluate the effect of the visual stimulation.
To evaluate differences in CSF-mobility, FA, ROI volume and patient information between CAA and healthy controls, Mann-Whitney U-tests were performed.

Specify type of analysis: ☐ Whole brain ☐ ROI-based ☒ Both

Anatomical location(s)	For study 1, the visual cortex ROI was defined based on the template z-score output (four datasets of study 1 did not contain a visual stimulation scout scan). A threshold of z-score>3.5 was used for all subjects of this study. Only the largest cluster of contiguous voxels was included in the mask. As the resolution of the fMRI scan used to create the z-score map was much lower than that of the CSF-STREAM, the obtained area not only contained the visual cortex but also the CSF in its vicinity.<br>ROIs were manually delineated using anatomical landmarks on the non-motion-sensitized CSF-scan (cf. Fig. 2) using ITK-Snap, as follows: The 4th ventricle ROI was drawn over 13 transversal slices; The ROI delimiting the SAS around the MCA was drawn over seven transversal slices on the left MCA branch;The motor cortex SAS sulci ROI was drawn over ten transversal slices; PVS in the basal ganglia were identified over sagittal slices as CSF-filled spaces around the lenticulostriate arteries; The ROI of PVS surrounding penetrating arteries in the white matter was drawn over 25 transversal slices in the centrum semiovale, starting from the slice directly above the lateral ventricles; The blood ROI was drawn inside one MCA branch over two slices; The noise ROI was drawn outside the brain, on the two central sagittal slices and on the corners of the same slice where the SAS MCA ROI was drawn.<br>To extract the final areas of interest, the manually delineated ROIs and the visual cortex ROI were multiplied with a CSF-mask, thus including only voxels containing CSF and not noise. This CSF-mask was created by first thresholding the non-motion-sensitized CSF-scan using a threshold of 150 (a.u.). After visual inspection, this threshold could be adapted individually to assure proper selection of PVS. For study 1, if a voxel had a CSF-mobility change higher than 50% in one or more of the cardiac/respiration/random datasets, it was excluded from the mask. Voxels that had no included neighboring voxels ("lonely" voxels) were also excluded from the mask. For the two PVS ROIs, an additional Frangi filter (0.6<σ<1 with a step of 0.2, Frangi vesselness constant = 0.5) was applied in order to ensure the exclusive inclusion of vessel-like structures and exclusion of noise.<br><br>For study 2, a visual stimulation scout scan was available for all subjects, and we therefore used individual z-score maps to create the visual cortex ROIs. A threshold of z-score>7 was used to define the visual area and a threshold of z-score<1 was used to define a control region (rest of the brain). For the visual cortex ROI, only the largest cluster of contiguous voxels was included in the ROI. Next, to ensure only voxels containing CSF were included and not noise, a mask based on the non-motion-sensitized CSF-scan was created using a threshold of 150. Voxels for which the CSF-mobility change between the 2 conditions (stimulation ON and OFF) was higher than 50% were considered as noise and excluded from the ROI. As the resolution of the fMRI scan used to create the z-score map was much lower than that of the CSF-STREAM, the obtained area not only contained the visual cortex but also the CSF around the visual cortex.<br><br>For the CAA cohort study, the CSF-STREAM sub-scans were first interpolated from a 0.50 mm to a 0.17 mm isotropic resolution and co-registered using Elastix. Subsequently, the mean eigenvalue of a rank-two positive definite tensor was computed (DTI post-processing) using Python 3.10 to assess CSF-mobility and FA. To minimize the effects of background noise, a cut-off value of 0.15 mm^2/s was used to exclude all voxels with unphysiologically high CSF-mobility values.<br>SAS-MCA segmentations: the M1 segment of the middle cerebral artery was first segmented semi-manually using anatomical landmarks on the non-motion-sensitized CSF-scan using MITK version v2022.10. The MCA-segmentation was then inflated using the imdilate Matlab function to create a CSF-mask containing the SAS around the MCA; inflation was done from 0.17 mm up to 3.00 mm with a step-size of 0.17 mm. To ensure only CSF-signal was selected in the mask, voxels with low CSF-signal in the non-motion sensitized scan were excluded.<br>PVS segmentations: Parcellated atlases from the T1-scan were generated using Freesurfer (Version 6.0). White matter segmentations, derived from the Freesurfer parcellation, were manually corrected if necessary. To capture comparable ROIs of the CSO in all participants, the eyes and the optic chiasm served as anatomical landmarks for reference plane definition. The dimensions of the CSO segmentation encompassed the entire white matter above the lateral ventricles. PVS within the defined CSO segmentation were semiautomatically segmented using a Meijering filter-based approach with a global threshold on the interpolated non-motion sensitized scan of CSF-STREAM (0.17 mm isotropic).

Statistic type for inference<br>(See Eklund et al. 2016)	n.a
Correction	n/a

Models & analysis

n/a	Involved in the study
☒	☐ Functional and/or effective connectivity
☒	☐ Graph analysis
☒	☐ Multivariate modeling or predictive analysis
