# Supplementary material for: Region-specific drivers of CSF mobility measured with MRI in humans
Source: Nat Neurosci. 2025 Oct 14;28(11):2392–401. doi: 10.1038/s41593-025-02073-3 (PMC12586159; doi:10.1038/s41593-025-02073-3)
Supplement: Supplementary file 3 — High-resolution, whole-brain CSF signal. Whole-brain CSF signal measured using the non-motion-sensitized reference scan, shown in one individual. [file 41593_2025_2073_MOESM3_ESM.pptx]

## Slide 1
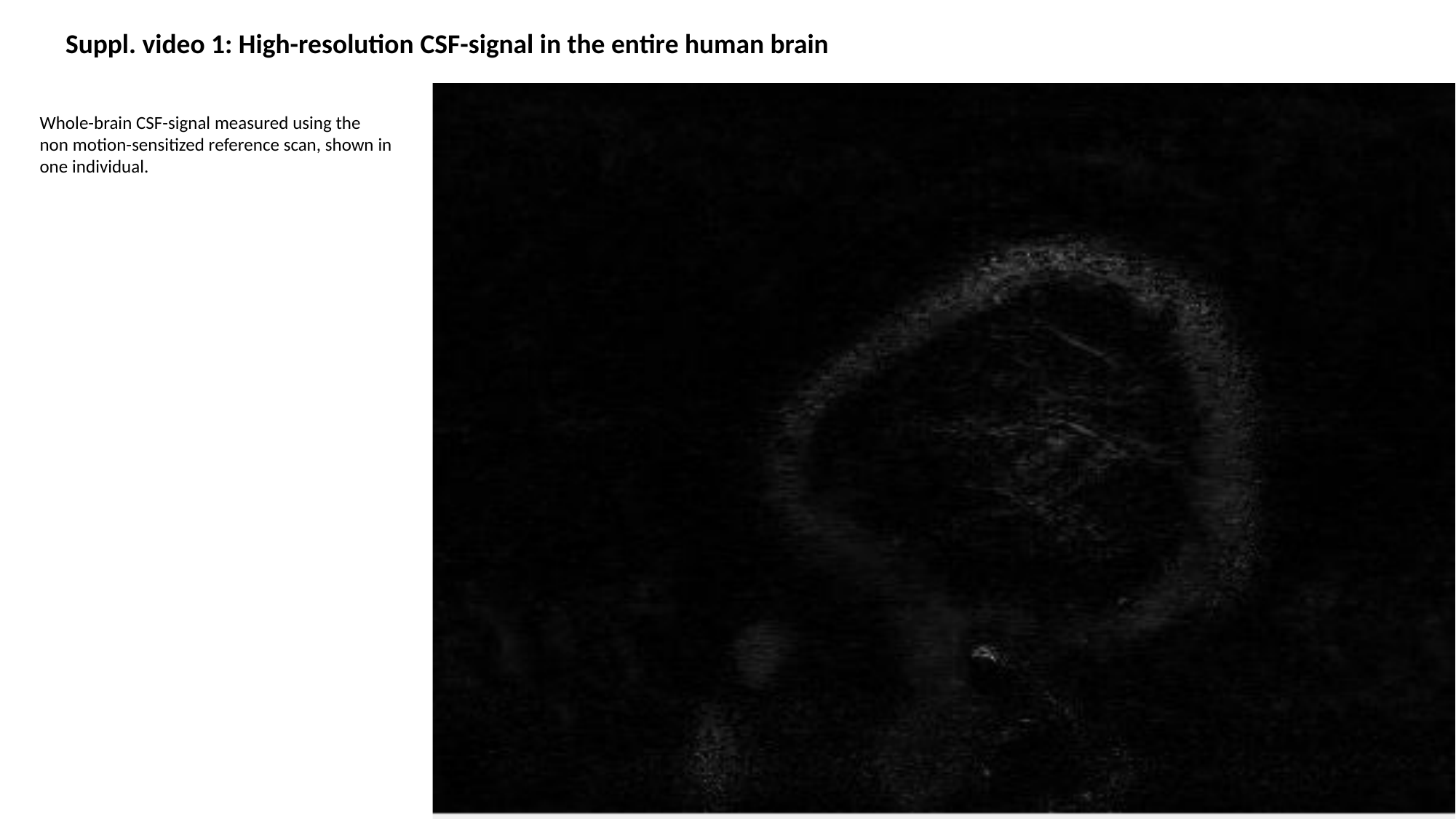

Suppl. video 1: High-resolution CSF-signal in the entire human brain
Whole-brain CSF-signal measured using the non motion-sensitized reference scan, shown in one individual.
CSF-signal
