# Supplementary material for: Region-specific drivers of CSF mobility measured with MRI in humans
Source: Nat Neurosci. 2025 Oct 14;28(11):2392–401. doi: 10.1038/s41593-025-02073-3 (PMC12586159; doi:10.1038/s41593-025-02073-3)
Supplement: Supplementary file 5 — Animation of CSF mobility change across driving forces in PVS around penetrating vessels. CSF mobility change from the mean value over phases (in %) across the cardiac (left), respiration (middle) and random (right) cycles in one representative individual (same data as in Fig. 4, but as a gif). Please note that a linear interpolation was applied between the phases to visually smoothen the video. [file 41593_2025_2073_MOESM5_ESM.pptx]

## Slide 1
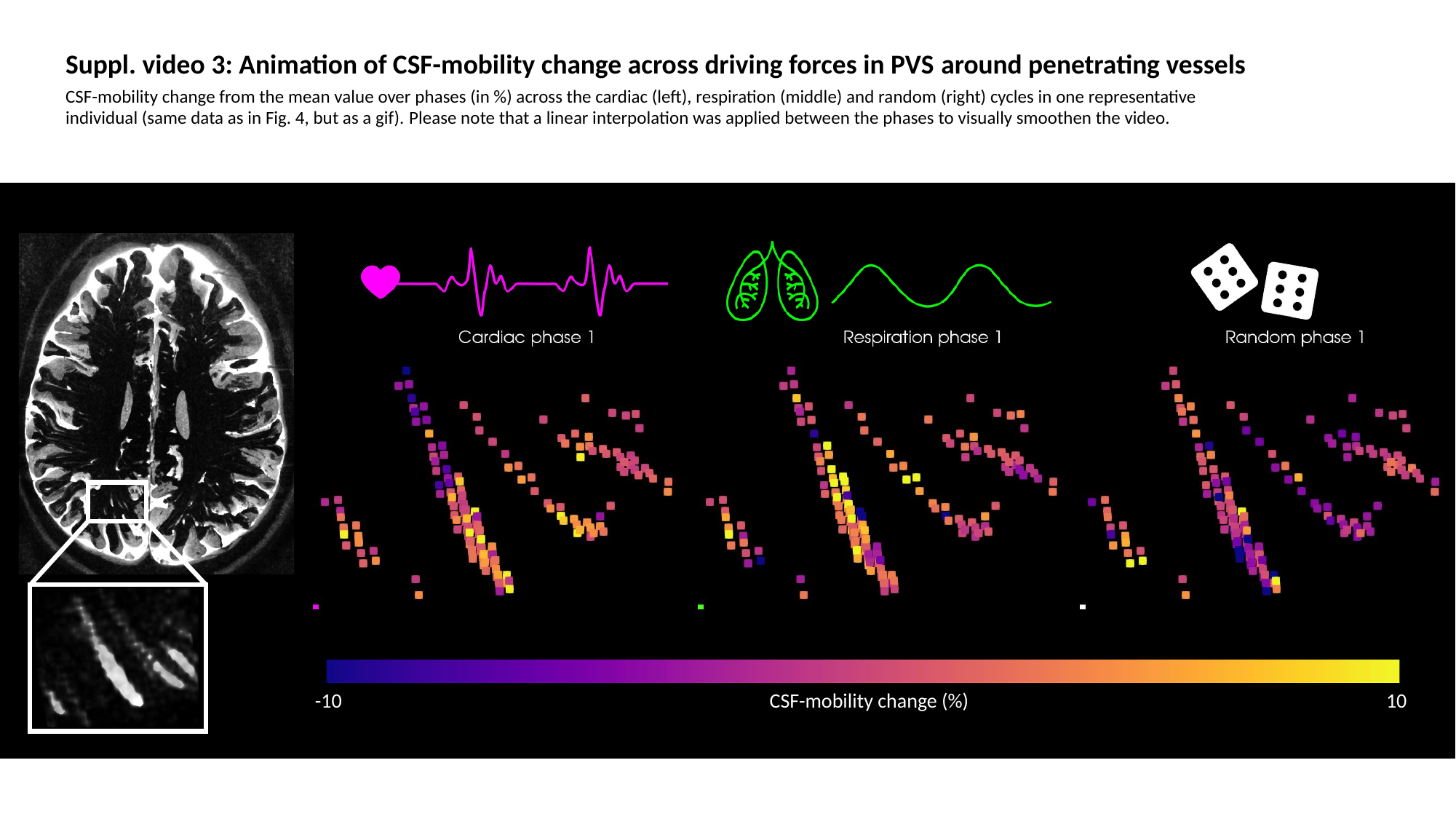

Suppl. video 3: Animation of CSF-mobility change across driving forces in PVS around penetrating vessels
CSF-mobility change from the mean value over phases (in %) across the cardiac (left), respiration (middle) and random (right) cycles in one representative individual (same data as in Fig. 4, but as a gif). Please note that a linear interpolation was applied between the phases to visually smoothen the video.
-10
CSF-mobility change (%)
10
